# Microbiomes of different ages in Rendzic Leptosols in the Crimean Peninsula

**DOI:** 10.7717/peerj.10871

**Published:** 2021-02-18

**Authors:** Anastasiia K. Kimeklis, Grigory V. Gladkov, Aleksei O. Zverev, Arina A. Kichko, Evgeny E. Andronov, Elena I. Ergina, Igor V. Kostenko, Evgeny V. Abakumov

**Affiliations:** 1 Applied Ecology, St. Petersburg State University, Saint-Petersburg, Russia; 2 Laboratory of Microbiological Monitoring and Bioremediation of Soils, All-Russian Research Institute for Agricultural Microbiology, Pushkin, Russia; 3 Genetics and Biotechnology, St. Petersburg State University, Saint-Petersburg, Russia; 4 V.V. Dokuchaev Soil Science Institute, Moscow, Russia; 5 V.I. Vernadsky Crimean Federal University, Simferopol, Russia; 6 Nikitsky Botanical Garden –National Scientific Center, Yalta, Russia

**Keywords:** Soil microbiome, 16S rRNA library sequencing, Rendzic Leptosol, Pedogenesis, Chronosequence, Soil liming

## Abstract

Rendzic Leptosols are intrazonal soils formed on limestone bedrock. The specialty of these soils is that parent rock material is more influential in shaping soil characteristics than zonal factors such as climate, especially during soil formation. Unlike fast evolving Podzols due to their leaching regime, Leptosols do not undergo rapid development due to the nature of the limestone. Little is known how microbiome reflects this process, so we assessed microbiome composition of Rendzic Leptosols of different ages, arising from disruption and subsequent reclamation. The mountains and foothills that cover much of the Crimean Peninsula are ideal for this type of study, as the soils were formed on limestone and have been subjected to anthropogenic impacts through much of human history. Microbiomes of four soil sites forming a chronosequence, including different soil horizons, were studied using sequencing of 16S rRNA gene libraries and quantitative PCR. Dominant phyla for all soil sites were Actinobacteria, Proteobacteria, Acidobacteria, Bacteroidetes, Thaumarchaeota, Planctomycetes, Verrucomicrobia and Firmicutes. Alpha diversity was similar across sites and tended to be higher in topsoil. Beta diversity showed that microbiomes diverged according to the soil site and the soil horizon. The oldest and the youngest soils had the most similar microbiomes, which could have been caused by their geographic proximity. Oligotrophic bacteria from Chitinophagaceae, Blastocatellaceae and Rubrobacteriaceae dominated the microbiome of these soils. The microbiome of 700-year old soil was the most diverse. This soil was from the only study location with topsoil formed by plant litter, which provided additional nutrients and could have been the driving force of this differentiation. Consistent with this assumption, this soil was abundant in copiotrophic bacteria from Proteobacteria and Actinobacteria phyla. The microbiome of 50-year old Leptosol was more similar to the microbiome of benchmark soil than the microbiome of 700-year old soil, especially by weighted metrics. CCA analysis, in combination with PERMANOVA, linked differences in microbiomes to the joint change of all soil chemical parameters between soil horizons. Local factors, such as parent material and plant litter, more strongly influenced the microbiome composition in Rendzic Leptosols than soil age.

## Introduction

The soil microbiome is an essential part of the soil structure (Attwood et al., 2019; Dubey et al., 2019; Wei et al., 2019). Understanding soil microbiome composition and function help reveal key processes of soil formation and implementation of vital ecosystem services (Doula & Sarris, 2016; Saleem, Hu & Jousset, 2019). The process of soil formation, or pedogenesis, depends on multiple factors, including climate, vegetation, topography, and parent material (Dokuchaev, 1883). The type of the parent material determines the rate of soil profile differentiation (Gagarina, Khantulev & Chikhikova, 1981; Gagarina, 1996), thus affecting microbiome formation. Hard limestone rock as a parent material promotes the formation of weakly developed soils, called Rendzic Leptosols (Homolák et al., 2017). Such soils are considered to be intrazonal, because local factors, such as parent material, affect their characteristics much more than climate (Perkins & Gettys, 1951). Limed soils have higher microbial biomass than unlimed (Bakina et al., 2014; Narendrula-Kotha & Nkongolo, 2017). Soil liming also affects the stability of humic acids, reducing labile humic content (Bakina et al., 2014). However, it does not affect organic matter content. Actinobacteria and Acidobacteria are more prevalent in more acidic soils with high carbon content and leaching of nitrates, while in less acidic soils with lower carbon content, nitrogen is accumulated, encouraging the growth of Proteobacteria (Bárta & Tahovská, 2017).

According to Targulian, every disruption of the soil surface sets soil formation process, or pedogenesis, to zero (Targulian & Bronnikova, 2019). Thus, different stages of the pedogenesis can be approached by studying chronosequences, which are series of soils, formed at different times under similar climatic and biogenic conditions (Emmer, 1995; Mokma, Yli-Halla & Lindqvist, 2004; Cerli et al., 2008; Abakumov et al., 2010). Soil chronosequences form at the terraces of reservoirs, on the dunes, under the barrows and the quarry dumps (Gennadiev, 1990). Series of coastal bars in Lake Ladoga (Russia), formed by a gradual lowering of the water level showed that in the process of pedogenesis, bulk soil is divided into horizons, and microbiome composition is divided according to these horizons (Ivanova et al., 2020a). Other objects used to evaluate pedogenesis are soils on reclaimed mining heaps (Anderson, 1977; Frouz, 2014; Sokolov et al., 2015). Initially, microbiomes of young soils are abundant in Chloroflexi and Cyanobacteria, photosynthetic bacteria which can survive with limited number of nutrients (Gladkov et al., 2019). Quite rapidly after development, however, copiotrophic bacteria populate these soils (Kimeklis et al., 2020).

The Crimean Peninsula contains numerous diverse climatic zones, ranging from the dry steppes in the north to the forest-steppe and forest in the mountains and subtropics on the southern coast (Lisetskii & Ergina, 2010). Origins, texture classes and chemical composition of parent material also have variation in different parts of the peninsula. Intensive human activity over thousands of years on the limestone formed differently aged soils on the calcareous parent material in this area (Dragan, 2005; Stolba, Lisetskii & Marinina, 2015). Moreover, open cast mining is the most severe type of current exogenic transformation of environments in the Crimean Peninsula. These parent materials are the most problematic in terms of ecosystem reclamation and restoration. Parent material alongside with topography constitute geogenic conditions, which determine the speed of soil formation (pedogenesis rate) (Brevik & Lazari, 2014). The role of parent materials in soils formation is directly connected with degree of consolidation and mineralogical composition, while the topography seriously effects the insolation rate and the degree of water retention capacity in elevated forms of relief (Targulian & Krasilnikov, 2007). In this context, the soils of the first two ridges of Crimea mountains represent well drained calcaric polypedones covered with Leptosols (or Lithosols) with weak profiles, not essentially differentiated in vertical scale. Thus, the chronosequences of soils in conditions of Crimea are less explored in sense of soil profiles developments rate in comparison with soil series of humid climate, located on acid or neutral parent materials. While in taiga zone 100-200 years is enough for development of embryonic soil profile, in case of Crimean forest-steppes of the mountain ridges the zonal soil profile normally forms 4–7 times longer.

Here we address the subject of microbiome composition in soils of different ages in multiple Rendzic Leptosols horizons of the Crimean forest-steppe zone. The subject of this study were four territories, formed under the same climatic conditions and from the same parent rock material, composing a chronosequence. Their age ranged from native soil to 700, 70 and 50 years, the range resulting from different anthropogenic impacts (Lisetskii & Ergina, 2010). The aim of this study was to investigate microbiome diversity, including bacteria and archaea, of the soil chronosequence on derivatives of limestones in different stages of ecosystem development, using quantitative PCR and high-throughput sequencing of 16S rRNA gene libraries. Investigation of these chronosequences may provide new information about the rates of soil formation during different stages of ecogenesis on the surface of limestones.

## Materials & Methods

### Study sites and sample collection

**Figure 1:**
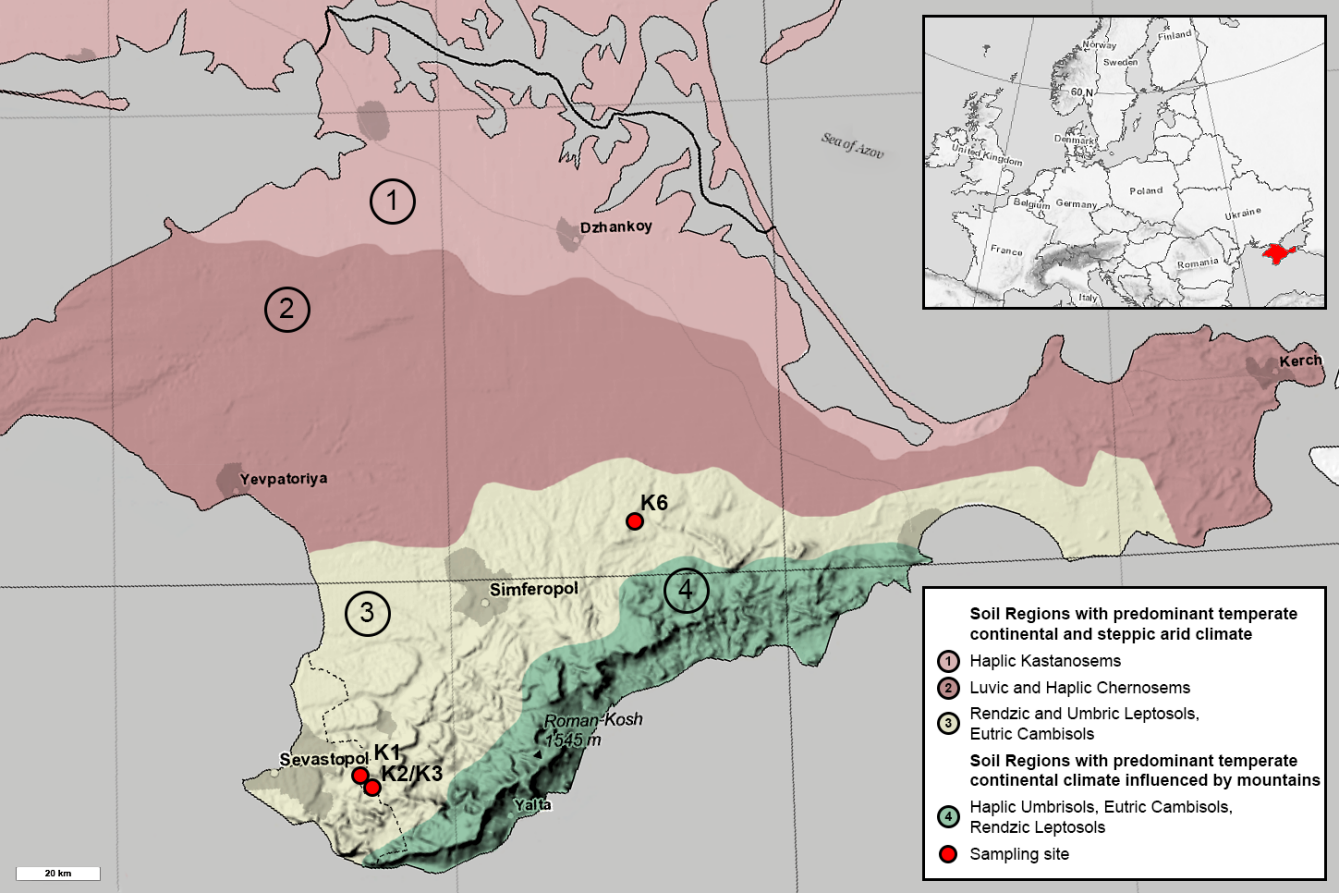
Map of the Crimean Peninsula and the location of sampling sites. Modified after Soil Regions Map of the European Union and Adjacent Countries (BGR, 2005). Colour and numbers 1-4 mark different soil types. Sampling sites are marked with red circles.

All sites are represented by the Rendzic Leptosols located in the first and second mountain ridges in the forest-steppe zone of Crimean Peninsula. The climate of this zone is more humid than in northern part of the Peninsula. The annual precipitation rate is about 380–500 mm per year while the evaporation rate is 750–850 mm. Annual average temperature is +20–22 °C. The depth of soil freezing is no more than 20 cm. Overall, the climate of the investigated area is very close to Mediterranean one. The heights of the relief range between 300–750 m, while topography of the territory is strongly affected by composition and texture of limestones. Limestones are presented by sedimentary rocks strongly affected by karst and denudation processes. Originally, limestone surface was not covered by any other quaternary sediment and this fact provides the possibility for the soil to be formed according to the model of primary soil formation. Thus, all sites are comparable in terms of pedogenesis conditions. Meanwhile, all sites comprise different chronosequence stages, which originated from anthropogenic exploitation of mines for construction and other processes in different historical periods. Age of each chronosequence stage was confirmed by historical documents (Lisetskii & Ergina, 2010). Benchmark site K3 was presented by native brown soil, formed around the Holocene. Site K1 with the oldest anthropogenic impact is located in the 700-year-old territory of medieval fortress city Eski-Kermen, which was destroyed at the end of the 14th century. Near K3 site is site K2, representing 75-year-old WWII trenches in Holmovka village. Site K6 is an overgrown quarry in the north of Belogorsky district with gravel-sandy textured carbonate containing heaps, which was reclaimed approximately 50 years ago. All soil profiles are Leptosols of various thicknesses; the thickness of the humus horizon and the degree of weathering of the fine earth soil increased with age. Samples were collected in the summer of 2018. They were taken from each soil profile for each horizon in 5 replicates. Quantity of horizons differed through sites due to differences in soil profiles: O, AY and C from the K1, AY and C from K2 and K3, AY from K6. The coordinates of the K1 site were 44°36.554 N, 33°44.376 E; K2 and K3 sites 44°39.171 N, 33°44.968 E; K6 site 45°07.644 N, 34°35.537 E (Fig. 1). All soil samples were acquired with the approval of V.I. Vernadsky Crimean Federal University.

Soils for routine analyses were ground and passed through a 2 mm sieve; the large root debris was removed manually. The main agrochemical parameters were measured: P_2_O_5_ and K_2_O by the Machigin method (GOST 26205-91, 1991), pH (GOST 26213-91, 1991) and total nitrogen (GOST 26107-84, 1984). Total organic carbon (TOC) was determined using a CHN analyser Leco CHN-628 (Leco Corporation, USA) in Research Park of St Petersburg State University.

### DNA isolation, real-time and 16S rDNA library preparation

For the microbiome analysis, five replicate soil samples were collected from each horizon from each site (40 total samples). From each sample, total DNA was isolated from 0.5 gram of soil using the NucleoSpin®Soil Kit (Macherey-Nagel GmbH & Co. KG, Germany) using a combination of SL1+SX buffers, recommended for soils with low organic content (Lazarevic et al., 2013). Samples were mechanically disintegrated using a Precellys 24 homogeniser (Bertin Technologies, France). The quality of isolation was tested by gel electrophoresis in 1% agarose gel (0.5 × TAE buffer). DNA concentrations were measured at 260nm using SPECTROstar Nano (BMG LABTECH, Ortenberg, Germany). The final DNA concentration was, on average, 50 ng/μL.

Quantitative PCR (qPCR) was conducted for two groups of organisms: bacteria and archaea as previously described in Gladkov et al. (2019). Each sample, including standards, was analysed in triplicate. The mean values with standard errors were calculated for replicates of both PCR and DNA samples. After processing, the results were expressed as the common logarithm of the number of ribosomal operons per 1 g soil.

Construction and sequencing of the 16S rRNA amplicon libraries was conducted using an Illumina MiSeq (Illumina, Inc, USA) at the Centre for Genomic Technologies, Proteomics and Cell Biology (ARRIAM, Russia) as described in Gladkov et al. (2019).

### Data processing

Amplicon libraries of the 16S rRNA gene were processed using packages in the R (R Core Team, 2018) and QIIME2 (Bolyen et al., 2019) software environments. RStudio Team (2016) was used as the development environment for R. Trimming, combining sequences into phylotypes and subsequent processing was performed through dada2 package (Nearing et al., 2018), which provides more reproducible and accurate results due to the use of denoising algorithms rather than clustering of phylotypes, in contrast to more classical approaches (Callahan et al., 2016). The taxonomic affiliation of phylotypes was determined using the RDP classifier (Wang et al., 2007) based on Silva 132 (Quast et al., 2013). The phylogenetic tree was built in the QIIME2 software environment using the SEPP package (Janssen et al., 2018). For some analyses, data were normalised by phyloseq (McMurdie & Holmes, 2013) using the rarefaction algorithm according to the sample with the smallest number of readings, and were stabilised by variation through the Deseq2 package (Love, Huber & Anders, 2014) to compare the relative abundances of phylotypes in the samples. For the analysis of alpha diversity, the following indices were used: the observed OTU, Shannon (Shannon & Weaver, 1949), the inverse Simpson (Simpson, 1949) and Faith phylogenetic diversity (Faith, 1992). Significance of mean differences was calculated by the Mann–Whitney test (Mann & Whitney, 1947). For the analysis of beta diversity, communities were compared using the construction of their dissimilarity matrix using the weighted UniFrac, unweighted UniFrac (Lozupone & Knight, 2005) and Bray-Curtis algorithms (Bray & Curtis, 1957). When visualising the data on beta diversity, the dimensions of the dissimilarity matrices were reduced using NMDS (Kruskal, 1964). The significance of sample separation in the analysis of beta diversity was assessed by PERMANOVA (Anderson, 2017) in the form of the adonis2 test as part of the vegan package (Oksanen et al., 2019). To analyse the variation of beta diversity by soil chemical parameters, the constrained correspondence analysis (CCA) was used (Ter Braak, 1986; Palmer, 1993; McCune, 1997). To assess the possible multicollinearity of the CCA model, generalised variance-inflation factors for linear models were used (Fox & Monette, 1992; Fox, 1997). The CCA function and reliability analyses of the model were conducted using the vegan package. To estimate the significance of differences between phylotypes, previously normalised data were processed using the Wald test, with a Benjamin-Hotchberg false discovery rate (FDR) correction in the DEseq2 package (Benjamini & Hochberg, 1995).

The R packages phyloseq, ggpubr (Kassambara, 2019), picante (Kembel et al., 2010), ggforce (Pedersen, 2019), tidyverse (Wickham et al., 2019), ggtree (Yu et al., 2018), ampvis2 (Andersen et al., 2018) were used for post-processing and visualisation of the obtained data.

## Results

### Soil chemical parameters

All soils demonstrated alkalinity (from 8.2 to 7.6) and a high content of carbonates (4.8–45.6%), which is typical of Rendzic Leptosols. For the K1 and K2 sites, pH and carbonates decreased towards the upper horizons (topsoil), due to leaching processes. Carbonate content in the C horizon at the K3 site (4.8%) was lower than in the AY horizon (28.57%) because most of the carbonates are immobilised in the skeleton of the soil. K1 was the only site with an O horizon in a soil profile; this type of horizon is formed by herbage without grazing. Therefore, it had the highest amount of total organic carbon (TOC) and nitrogen content. Leptosol in site K6 was of a slightly alkaline pH (7.7) and had significant reserves of potassium (1,110 mg/kg) and phosphorus (285 mg/kg), caused by the use of the surrounding territory by the locals of the Vishennoe village to dispose of household waste (Table 1).

### Quantitative PCR

**Figure 2:**
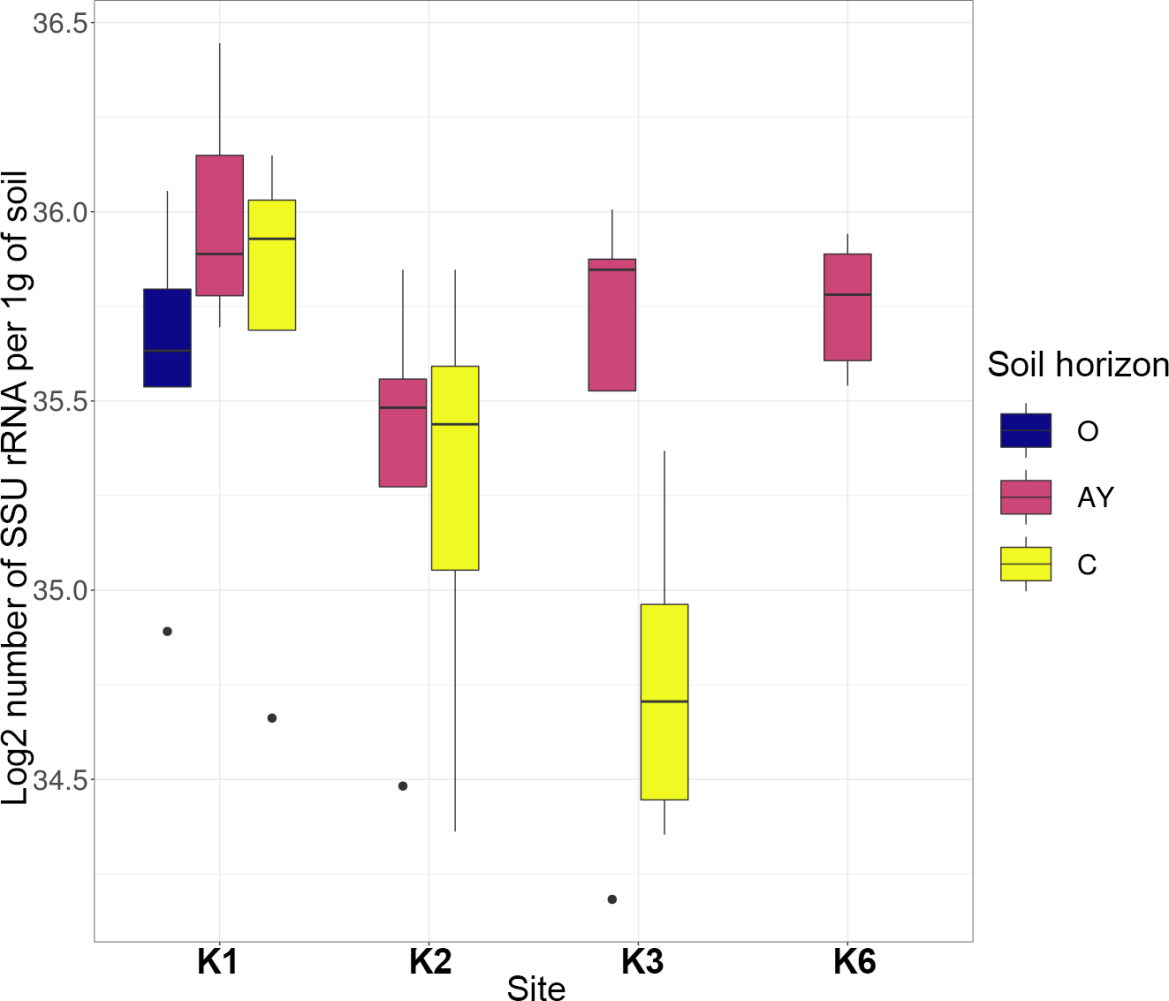
Abundance of bacteria and archaea in all samples assessed by qPCR. Values are given as the common logarithm of the means of ribosomal operon number per 1g soil (*n* = 15). Significance is given as a standard error of means.

Quantitative PCR showed that the number of bacteria ribosomal operons per 1g of soil was high across all sites and horizons (Fig. 2). The archaea operon count varied by horizon, but for K1 and K2 sites, it increased towards the lower horizons.

### Initial quality control and phyla composition

After initial processing of 40 amplicon libraries of 16S rRNA genes, three samples were excluded from the subsequent analysis due to their poor agreement with a rarefaction curve (Fig. S1). All data is available at SRA database (SRA Toolkit Development Team, 2020) under BioProject ID PRJNA645404. The final output of the 16S rRNA gene library sequencing included 37 samples with a total of 1145454 reads. The minimum number of detected reads were 13,925, maximum – 41,384 and average read number – 30,958.22. A total of 12,311 OTUs were observed: 11,705 (95%) OTUs were assigned to the kingdom level, 11,026 (89.56%) –phylum level, 10,814 (87.84%) –class level, 9406 (76.4%) –order level, 7800 (63.36%) –family level, 3993 (32.43%) –genus level and 277 (2.25%) –species level.

**Table 1:** Main soil chemical parameters.

Site	Description	Horizon	P_2_O_5_ (mg/kg)	K_2_O (mg/kg)	pH	TOC (%)	C_carb_ (%)	N_tot_ (%)
K1	Eski-Kermen. 700 years	O	123	515	7.6	>22.95	20.24	1.47
		AY	12	212	8.0	7.32	34.50	0.78
		C	6	45	8.2	0.23	33.12	0.03
K2	Holmovka. 75 years	AY	8	595	7.9	6.84	34.13	0.10
		C	2	14	8.2	0.47	45.60	0.43
K3	Holmovka. Benchmark soil	AY	11	820	7.8	8.88	28.57	0.48
		C	5	56	8.1	0.67	4.80	0.05
K6	Leptosol. 50 years	AY	285	1110	7.7	11.70	23.81	0.58

**Figure 3:**
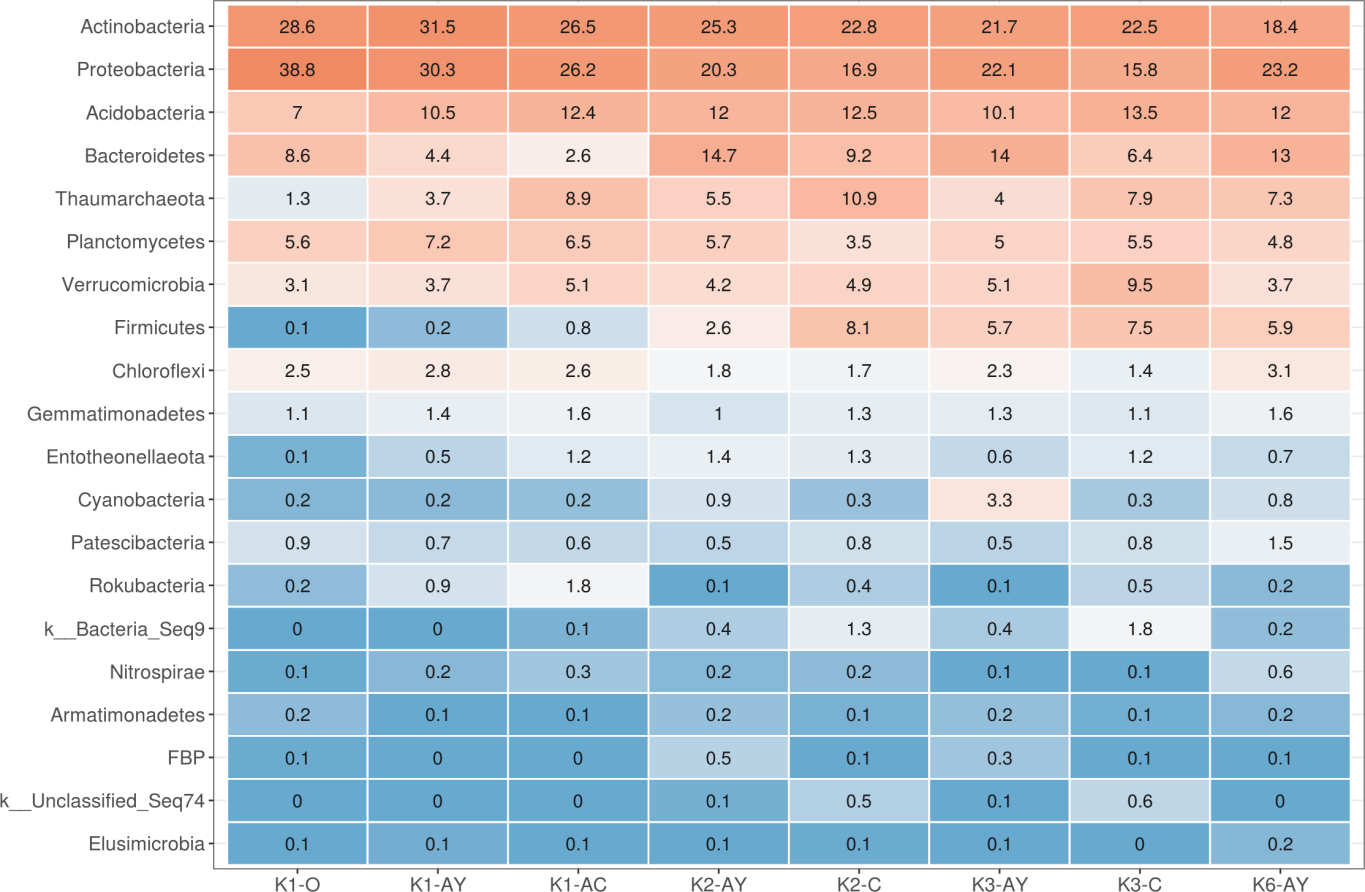
Heatmap for the 20 most abundant phyla across all samples. Orange stands for more abundant, and blue for less abundant.

The most abundant phyla across all samples were Actinobacteria, Proteobacteria, Acidobacteria, Bacteroidetes, Thaumarchaeota, Planctomycetes, Verrucomicrobia, Firmicutes and Chloroflexi (Fig. 3). Site K1 was most clearly distinct from other sites by phyla composition, the most drastic difference being the almost complete absence of Firmicutes representatives. Some phyla demonstrated shifts in abundance correlating with the soil horizon: Bacteroidetes and Proteobacteria were more abundant in topsoil horizons, while Thaumarchaeota, Acidobacteria and Verrucomicrobia were more abundant in lower horizons. This observation is consistent with qPCR data. Bacterial ribosomal count was approximately equal between horizons probably because different bacteria groups shift their abundance in opposing directions through the horizons; and Thaumarchaeota, being the dominant archaeal phyla, was responsible for total archaea increase in lower horizons.

On a family level, the most abundant taxa were Nitrososphaeraceae (Thaumarchaeota), Chitinophagaceae and Microscillaceae (Bacteroidetes), 67-14 and Micromonosporaceae (Actinobacteria), Xanthobacteriaceae and Burkhorderiaceae (Proteobacteria) and Pyrinomonadaceae (Acidobacteria) (Fig. S2). In K1 site samples, phylotypes from Rubrobacteriaceae and Bacillales were less abundant than in other samples and Solirubrobacteriaceae were more abundant. Sphingomonadaceae were more abundant in topsoil. Xiphinematobacteriaceae were more abundant in deeper AC and C horizons.

### Alpha diversity

**Figure 4:**
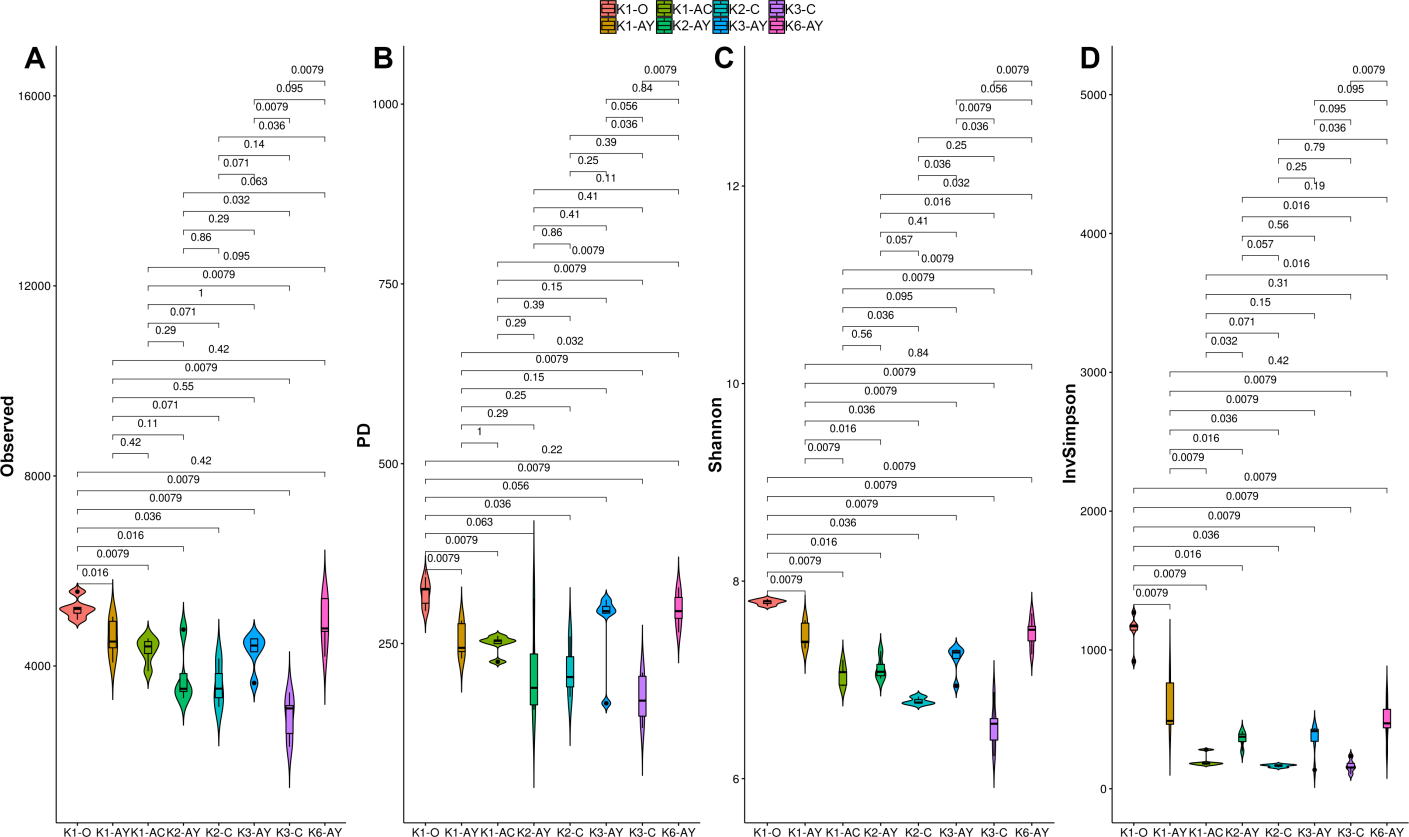
Alpha diversity indexes for each soil horizon. (A) Observed, (B) PD, (C) Shannon, (D) inverted Simpson. Data presented by violin and box plots, which show the kernel probability density of the data at different sample values. *P*-values are given above plots.

All alpha diversity indices revealed the higher horizons demonstrated a tendency towards higher diversity (Fig. 4). The maximum observed number of OTUs was detected in K1-O and K6-AY, and the minimum in K3-C. In the K1 and K3 sites, observed OTU significantly decreased toward the lower horizon. AY horizons across all sites had comparable OTU numbers. The Faith index, which demonstrates phylogenetic distance (PD), was evenly distributed between samples, with no apparent maximum or minimum. However, it also showed separation of samples by horizon. The Shannon index evaluates diversity, particularly evenness, with respect to minor taxa, while the inverted Simpson index takes into account more abundant taxa. Using the Shannon index, K1-O was similar in diversity to K6-AY but was different according to the inverted Simpson index. In general, the inverted Simpson index shows that K1-O was most diverse, while samples from K2, K3 and K6 sites show significant, but slight, separation from each other. Furthermore, K6-AY was closer in diversity to K1-O and K1-AY than K2 and K3 sites by the Shannon index. In summary, all diversity indices to varying extents show a separation of samples by soil horizon, alongside with secluded position of samples from K1 and K6 sites.

### Beta-diversity and CCA

**Figure 5:**
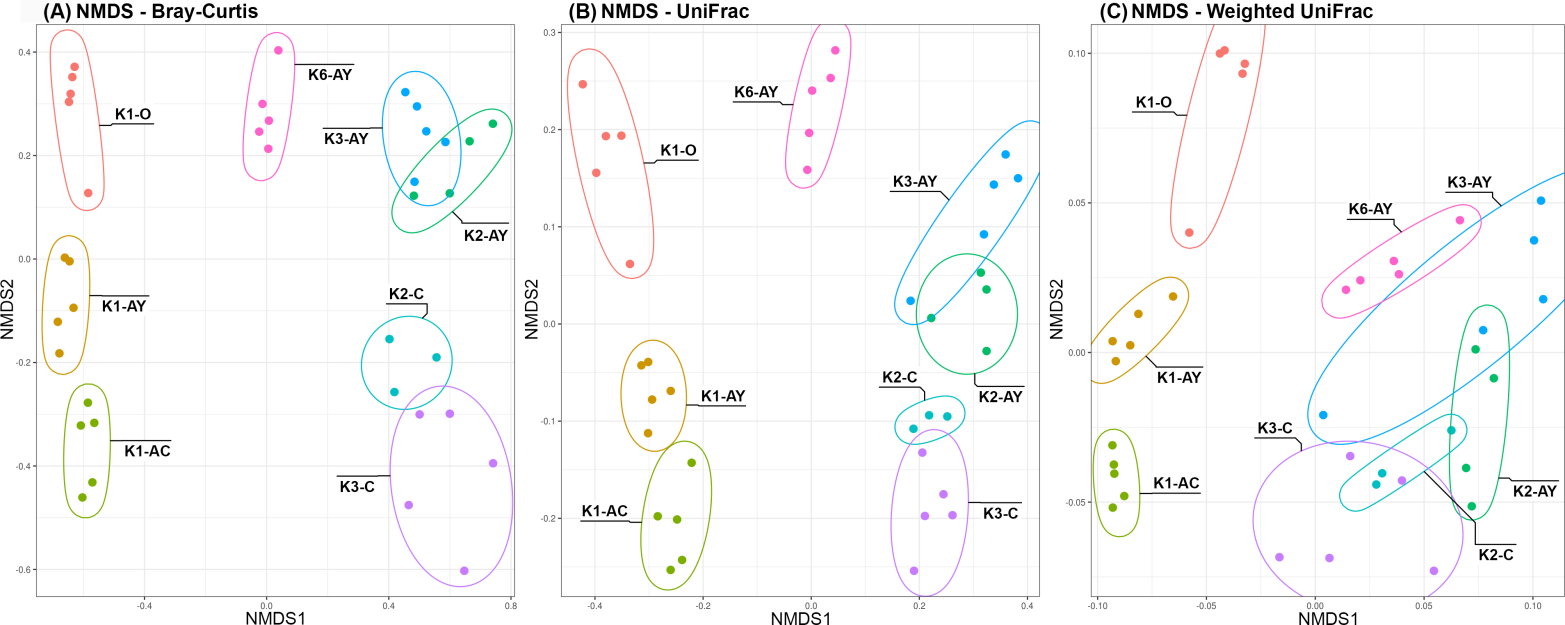
NMDS plots of Beta diversity. (A) Bray–Curtis distance matrix. (B) UniFrac. (C) weighted UniFrac. Sample repeats are surrounded by ellipses, estimated using the Khachiyan algorithm.

Beta diversity demonstrated two clear trends, coinciding with the axes (Fig. 5). Along the “Y” axis samples tended to line up according to the soil horizons. Along the “X” axis, samples were divided into “site” groups: Bray-Curtis and UniFrac algorithms showed that one group included all samples from the K1 site, the second group included the only sample from K6 site and the third, all samples from both K2 and K3 sites. According to the weighted UniFrac algorithm, samples from the K6 site group together with samples from K2 and K3 sites, which is consistent with results of the inverted Simpson index.

PERMANOVA showed that soil horizon had the maximum coefficient of determination (Table 2). The next factor was the sampling site. All the soil agrochemical parameters, except for carbonates (C_carb_), demonstrated similar significance, but with low coefficient of determination values. PERMANOVA nested by horizon showed that all agrochemical factors, including C_carb_, became significant (Table S1).

**Table 2:** Coefficient of determination (R2) for each soil factor assessed by PERMANOVA.

Factor	R^2^	Pr(>F)
Horizon	0.52179978	0.001
Site	0.49421618	0.001
N_tot_	0.18573955	0.001
TOC	0.17705733	0.001
pH	0.15795176	0.001
K_2_O	0.14989736	0.001
P_2_O_5_	0.11987081	0.002
C_carb_	0.04737008	0.100

**Figure 6:**
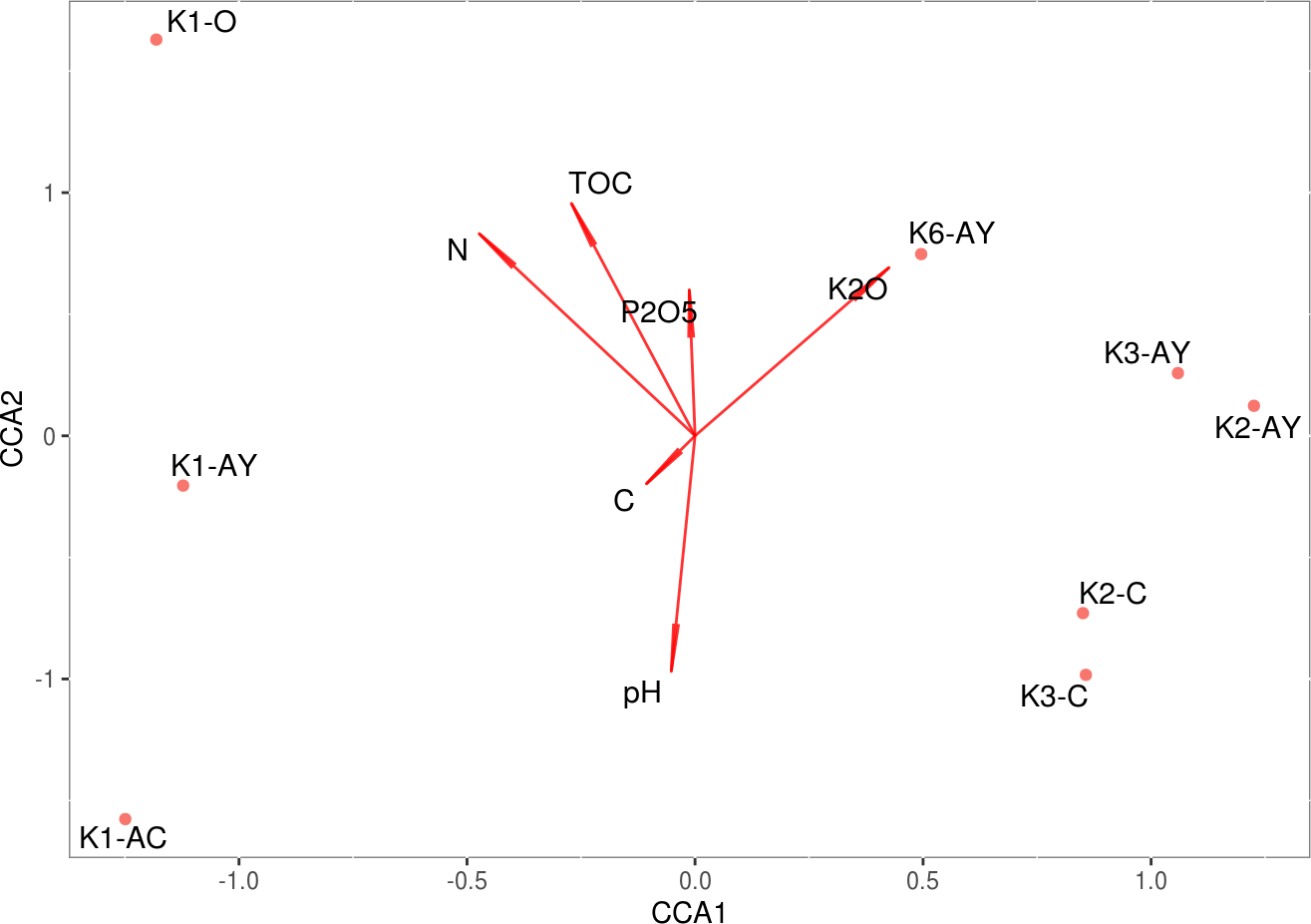
CCA. Direction of the vectors shows the degree of covariation between factors.

The model of CCA performed for agrochemical factors is statistically significant, although it demonstrated that these factors could not explain the discrepancy between sample sites (Fig. 6). However, they explained soil stratification into horizons. The test on the variance inflation factor showed all agrochemical factors, including pH, demonstrated multicollinearity. A combination of CCA and PERMANOVA confirm that variability between soil horizons was associated with agrochemical factors.

### K1/K3 phylotype comparisons

**Figure 7:**
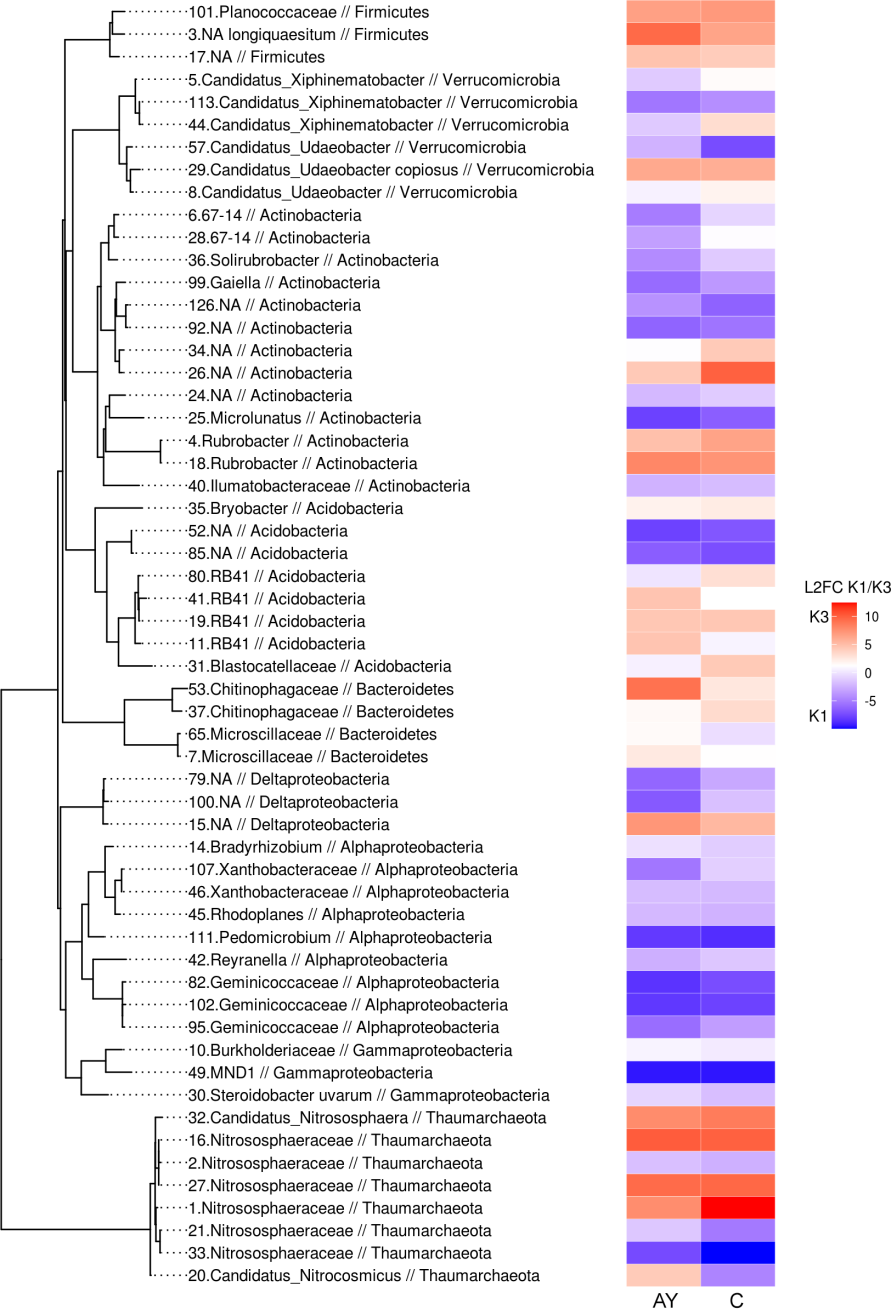
Phylogenetic tree with phylotypes, which abundance shifts significantly (padj < 0.05) between K1 and K3 sites. Shifts are presented as log2foldchange values. The left column shows shifts in the AY horizon, right column, the AC/C horizon. Red indicates an increase in K3, blue, in K1.

Previous analyses concluded that microbiomes across all sites separate by soil horizon, but also that microbiomes from the K1 site are more distinct from other sites. To assess more precise differences in microbiome composition between sites, we visualised significant shifts of phylotype abundance in AY and AC/C horizons between the K1 and K3 sites (Fig. 7). Despite the major trend of microbiome differences between soil horizons, our analysis shows that the reactive component of the soil microbiome shifted together in both soil horizons between different soil sites. Firmicutes, in particular Planococcaceae and *B*. *longiquaestium*, increased in K3; Actinobacteria (*Solirubrobacter*, *Gaiella*, 67-14, *Microlunatus*, Ilumatobacteraceae) mostly increased in K1, except for *Rubrobacter*; Proteobacteria (Deltaproteobacteria, *Bradyrhizobium*, Xanthobacteriaceae, *Rhodoplanes*, *Pedomicrobium*, *Reyranella*, Geminicoccaceae, Burkhordeliaceae, MND1, *Steroidobacter uvarum*) were more abundant in K1. Representatives of Verrucomicrobia (*Xiphinematobacter*, *Udaeobacter*), Thaumarchaeota (Nitrososphaeraceae) and Acidobacteria (NA, RB41) varied in both K1 and K3 sites. Variation of Thaumarchaeota in both K1 and K3, the growth of which depends on nitrogen content, supports the earlier conclusions that nitrogen content doesn’t explain site differences. However, the K1 site was abundant in Actinobacteria and Proteobacteria related phylotypes.

### K2/K3 phylotype comparisons

Microbiomes of the AY and C horizons from two sites in Holmovka village (K2 and K3) were the closest to each other on the beta diversity plots. These data are supported by log2FoldChange values for the 30 of the most abundant phylotypes of both horizons between sites (Table S2). Almost half of these phylotype changes were not significant. The greatest differences in topsoil (more than 10 times more at K3-AY than K2-AY) were for Seq13 (Oxyphotobacteria from Cyanobacteria), Seq101 and Seq136 (Planococcaceae from Firmicutes), Seq322 (Chitinophagaceae from Bacteroidetes) and Seq339 (*Romboutsia* from Firmicutes). For the deeper horizon, the only phylotype matching these conditions was Seq445 (*Adhaeribacter* from Bacteroidetes).

### K6/K3/K1 phylotype comparisons

To assess the specificity of microbiome composition of the Leptosol at the K6 site, similar to K2 and K3 sites, we estimated shifts in abundance by calculating log2FoldChange values for 30 phylotypes for K6-AY/K3-AY and K6-AY/K1-AY pairs (Table S3). All log2FoldChange values were significant, except for the only phylotype in the K6-AY/K3-AY pair. Eleven phylotypes appeared in both pairs of comparisons, and most of them were more abundant in K6-AY: Seq20 and Seq161 (Nitrososphaeraceae from Thaumarchaeota), Seq11 and Seq119 (RB41 from Acidobacteria), Seq94 (Subgroup 6 from Acidobacteria), Seq53 (Chitinophagaceae from Bacteroidetes) and Seq165 (Oxyphotobacteria from Cyanobacteria). However, a lot of other phylotypes were underrepresented in K6 compared to the other two sites. In comparison with K3-AY, the K6-AY site contained more than 10 times fewer of the following phylotypes: Seq13 (Oxyphotobacteria from Cyanobacteria), Seq5 (Candidatus_*Xiphinematobacter* from Verrucomicrobia), Seq37 (Chitinophagaceae from Bacteroidetes), Seq101 and Seq136 (Planococcaceae from Firmicutes), Seq60 (*Aridibacter famidurans* from Acidobacteria) and Seq34 (Thermoleophilia from Actinobacteria). Compared to K1-AY, the K6-AY site contained more than 10 times less of the following phylotypes: Seq6 and Seq36 (Thermoleophilia from Actinobacteria), Seq33 (Nitrososphaeraceae from Thaumarchaeota), Seq25 (*Microlunatus* from Actinobacteria) and Seq49 (MND1 from Proteobacteria). K6-AY was more abundant than K1-AY by Seq3 (*Bacillus longiquaesitum* from Firmicutes) and Seq32 (*Candidatus* _*Nitrososphaera* from Thaumarchaeota). These differences show that topsoil microbiomes of all sites were composed of similar major phylotypes, including both oligo- and copiotrophic taxa, which shifted between sites regardless of their trophic group. These data are consistent with the observation that the changing soil chemical parameters have not explained the beta diversity observed between sites.

## Discussion

In our comparisons we focused on differences between Leptosols of various ages. Microbiomes of all these soil samples shared some similar taxa at the phylotype level, but most of them shifted their abundance according to the soil site or soil horizon. One of the major groups of phylotypes was composed of archaea from the Nitrososphaeraceae family in the Thaumarchaeota phylum. These archaea are capable of ammonia oxidation and are considered to play a significant role in nitrogen cycling in the soil, especially in an arid and low-nutrition environment (Pester, Schleper & Wagner, 2011; Kimble et al., 2018; Nelkner et al., 2019). Consistent with this, we found Nitrososphaeraceae more frequently inhabited deep soil horizons that were poor in nutrients across all studied sites. Moreover, it was the least frequent in the O horizon at the K1 site, which was the richest in total nitrogen. Notably, microbiomes from each site had dominant Nitrososphaeraceae of different phylotypes, e.g., Seq1 was more abundant in K3, Seq2 in K1 and Seq16 in K6. However, this segregation of phylotypes did not affect the overall dominance of Nitrososphaeraceae across horizons at different sites (Fig. S2). It should be noted that high amount of archaeal phylotypes goes in concordance with high amount of archaea in samples shown by qPCR.

The second largest family across all sites was Chitinophagaceae from Bacteroidetes. Bacteroidetes are oligotrophs (Fierer, Bradford & Jackson, 2007). Representatives within this phylum, Chitinophagaceae in particular, are essential for carbon decomposition, especially in sandy, loamy soils (Ho et al., 2017; Fernandes et al., 2018). Consistent with this data, Bacteroidetes were more abundant in the lower nutrient soils of K2 and K3 sites.

The representatives of the phylum Acidobacteria are sensitive to soil acidity, macro- and micronutrients, capable of utilising nitrite and playing a role in cellulose decomposition (Kielak et al., 2016). They are also considered oligotrophs (Fierer et al., 2012). This phylum is one of the major ones in our dataset, but in comparison with previous data on soil microbiome composition (Janssen, 2006; Jones et al., 2009), its relatable abundance was quite low. At the first sight this is consistent with the fact that its representatives are usually linked to acidic environments (Belova et al., 2018; Ivanova et al., 2020b), while soils from our dataset are alkaline. However, acidobacteria are gram negative and very sensitive to drought (Barnard, Osborne & Firestone, 2013; Chodak et al., 2015; Zhou et al., 2016), so another explanation of low relative abundance of acidobacteria, in our dataset can be connected to the season of sample collection (summer) or microbiome alterations during sample transportation. For instance, representatives of Pyrinomonadaceae family, present in all samples, live in arid conditions and be able to utilise a limited spectrum of carbon and energy sources (Wüst et al., 2016). Sites K2 and K3 were abundant in Blastocatellaceae, whose members have been isolated from African Savannah soils with low nutrient contents and were reported to be able to degrade complex carbon compounds (Huber et al., 2017).

In contrast to Bacteroidetes and Acidobacteria, Proteobacteria (especially Alphaproteobacteria)—are considered to be mostly copiotrophs (Campbell et al., 2010; Ramirez et al., 2010; Fierer et al., 2012). As expected, Proteobacteria members were the most abundant in the most nutrient-rich soil of the K1 site. Xanthobacteraceae members, dominant in this dataset, demonstrate a variety of metabolic strategies, including aerobic chemoheterotrophy, facultative chemolithoautotrophy and nitrogen fixation (Kappler & Nouwens, 2013; Oren, 2014). Some also live in association with leguminous plants. Sphingomonadaceae are commonly isolated from the soil and rhizosphere in particular (Glaeser & Kämpfer, 2014). They are reported to be a possible tool of bioremediation due to their ability to degrade xenobiotic and recalcitrant (poly)aromatic compounds.

Actinobacteria is one of the essential bacteria groups in the soil, significantly contributing to the carbon cycle via their cellulolytic activity (Lewin et al., 2016), so they are usually associated with the rhizosphere (Oberhofer et al., 2019). It is the most abundant phylum in our dataset, but the least amount was detected in the Leptosol of the K6 site, which could mean that its vegetation cover is not yet restored. The Actinobacteria phylum has been shown to include both copio- and oligotrophic bacteria (Morrissey et al., 2016). Representatives of Rubrobacteriaceae family, found in the K2 and K3 sites, have been reported to be oligotrophic. Some studies have shown that these bacteria are also associated with lime wall paintings and painted statues of Maijishan Grottoes (Schabereiter-Gurtner et al., 2001; Duan et al., 2017).

One of the most abundant phyla, determined using standard microbiological approaches, was Firmicutes. However, sequencing of 16S amplicon libraries showed that this is not always the case (Janssen, 2006). Sometimes they comprise as low as 2% of the total soil microbiome. Meanwhile, spore-forming *Bacillus* was reported to be highly associated with the rhizosphere (Toyota, 2015). Firmicutes, as gram-positive bacteria, are very resistant to many adverse environmental conditions. They are also drought resistant. In our dataset Firmicutes is a minor phylum, appearing mostly in the lower horizons of K2 and K3 sites, and the topsoil of the K6 site.

Another major phylotype from our dataset belonged to *Xiphinematobacter*, a nematode symbiont (Brown et al., 2015). Interestingly, it was mostly found in AC and C horizons. It was likely an amplification artefact since deeper horizons of soil had much less DNA.

Despite age differences, soil microbiomes from the K2 and K3 sites were the closest to each other based on beta diversity. However, alpha diversity analysis revealed that the difference between horizons in K3 was more pronounced than in K2. Probably, disturbance of soil in K2 did not affect the composition of the microbiome, but facilitated its penetration into lower soil horizons. According to the results of weighted metrics (inverted Simpson index, weighted unifrac algorithm), the microbiome from the Leptosol of the K6 site is grouped with samples from the K2 and K3 sites. However, by the results of unweighted metrics (Shannon index, Bray-Curtis and unweighted unifrac), the K6 site diverged from other sites, which could indicate that the major microorganisms are similar in all these sites, but the K6 site has a significant portion of a minor microbiome component. Soil from the K1 site was the most unique of all sites, likely because it was under anthropogenic influence from the 6th to the 14th centuries (AD).

The structure of Rendzic Leptosol leads to a horizontal organisation, where the upper horizon contains high amounts of humic compounds, and between it and the rock lies a fine-earth transition horizon. In these conditions, it is reasonable to assume that microbiome composition would be significantly different between these horizons (Taş et al., 2018). Therefore, we tried to link diversity of microbiome composition to several factors, such as site, horizon and different agrochemical parameters. Beta-diversity showed that samples grouped according to both site and soil horizon. Combination of CCA and PERMANOVA revealed that the most significant factor for beta-diversity were the nutrients associated with the soil horizon. Although we could discern that the difference between microbiomes of different soil horizons was linked with the changing of all soil agrochemical parameters, all these parameters, including pH, shifted together, and it was impossible to identify the influence of any individual factor.

## Conclusions

Here we focused on a microbiome composition of differently aged Rendzic Leptosols. As they are intrazonal, these Rendzic Leptosols soils are profoundly affected by their parent material and undergo very slow paedogenic process. Our research demonstrated that soil type on a limestone rock is the driving force behind microbiome shaping, without any apparent influence of its age. Overall, microbiomes from all sites were deficient in Acidobacteria due to the alkalinity or aridity of the environment. The benchmark soil was rich in oligotrophic bacteria (Chitinophagaceae, Blastocatellaceae, Rubrobacteriaceae), able to decompose complex carbon sources. The youngest soil microbiome was the most similar to the benchmark, with only slight differences in microbiome diversity between horizons. Site K1 was the only one with topsoil, formed by plant litter. It introduced additional organic matter, thus promoting an increase in copiotrophic bacteria (Xanthobacteriaceae, representatives of Actinobacteria). Despite that, the major factor determining soil microbiome composition was the nutrients associated with the soil horizon, and our analysis showed that the reactive component of the soil microbiome shifted simultaneously in both soil horizons between different soil sites.

##  Supplemental Information

10.7717/peerj.10871/supp-1 Supplemental Information 1 R markdown with main data analysis

10.7717/peerj.10871/supp-2 Supplemental Information 2 Raw data for qPCR

10.7717/peerj.10871/supp-3 Supplemental Information 3 Plot with rarefaction curves Shows ratio of observed OTU (phylotypes) and depth of sequencing. Each colour represents all replicates from a certain soil horizon in a certain site.

10.7717/peerj.10871/supp-4 Supplemental Information 4 Heatmap for most abundant families across all samples Orange is more abundant, blue –less.

10.7717/peerj.10871/supp-5 Supplemental Information 5 Coefficient of determination (R2) nested by Horizon for each other soil factor assessed by PERMANOVA

10.7717/peerj.10871/supp-6 Supplemental Information 6 Shifts of major phylotype abundance between different pairs of samples, expressed by Log2FoldChange values. Significant values are given in bold (padj < 0,05).

## Additional Information and Declarations

### Field Study Permissions

The following information was supplied relating to field study approvals (i.e., approving body and any reference numbers): All soil samples were collected under permission of V.I. Vernadsky Crimean Federal University, Simferopol, Russia.

### DNA Deposition

The following information was supplied regarding the deposition of DNA sequences: Data are available at the SRA database: PRJNA645404.

### Data Availability

The following information was supplied regarding data availability: Raw data are available in the Supplementary File.
