# Supplementary material for: Microbiomes of different ages in Rendzic Leptosols in the Crimean Peninsula
Source: PeerJ. 2021 Feb 18;9:e10871. doi: 10.7717/peerj.10871 (PMC7897411; doi:10.7717/peerj.10871)
Supplement: Supplemental Information 1 [file peerj-09-10871-s001.html]

krim\_chrono.utf8


```
  title: "krim_chrono"
author: "GrGladkov"
date: "14 7 20"
output: html_document
```

### Data import from the standart DADA2 pipeline.

```
setwd("~/storage/krim_chrono/shit_data/")
library(phyloseq)
ps <- readRDS(file = "~/storage/krim_chrono/shit_data/ps.krim_chrono.rds") 
metadata <- ps@sam_data
metadata$Horizon <- as.character(metadata$Horizon)
metadata$Horizon[metadata$Horizon == "A"] <- "AY"
metadata$Horizon <- as.factor(metadata$Horizon)
metadata
```

```
##               Site Horizon Description P2O5  K2O  pH Organic Carbonates    N
## kimeklis.K.10   K1      AY       K1-AY   12  212 8.0    7.32      34.50 0.78
## kimeklis.K.11   K1      AC       K1-AC    6   45 8.2    0.23      33.12 0.03
## kimeklis.K.12   K1      AC       K1-AC    6   45 8.2    0.23      33.12 0.03
## kimeklis.K.13   K1      AC       K1-AC    6   45 8.2    0.23      33.12 0.03
## kimeklis.K.14   K1      AC       K1-AC    6   45 8.2    0.23      33.12 0.03
## kimeklis.K.15   K1      AC       K1-AC    6   45 8.2    0.23      33.12 0.03
## kimeklis.K.16   K2      AY       K2-AY    8  595 7.9    6.84      34.13 0.10
## kimeklis.K.17   K2      AY       K2-AY    8  595 7.9    6.84      34.13 0.10
## kimeklis.K.18   K2      AY       K2-AY    8  595 7.9    6.84      34.13 0.10
## kimeklis.K.19   K2      AY       K2-AY    8  595 7.9    6.84      34.13 0.10
## kimeklis.K.1    K1       O        K1-O  123  515 7.6   22.95      20.24 1.47
## kimeklis.K.20   K2      AY       K2-AY    8  595 7.9    6.84      34.13 0.10
## kimeklis.K.21   K2       C        K2-C    2   14 8.2    0.47      45.60 0.43
## kimeklis.K.22   K2       C        K2-C    2   14 8.2    0.47      45.60 0.43
## kimeklis.K.23   K2       C        K2-C    2   14 8.2    0.47      45.60 0.43
## kimeklis.K.24   K2       C        K2-C    2   14 8.2    0.47      45.60 0.43
## kimeklis.K.25   K2       C        K2-C    2   14 8.2    0.47      45.60 0.43
## kimeklis.K.26   K3      AY       K3-AY   11  820 7.8    8.88      28.57 0.48
## kimeklis.K.27   K3      AY       K3-AY   11  820 7.8    8.88      28.57 0.48
## kimeklis.K.28   K3      AY       K3-AY   11  820 7.8    8.88      28.57 0.48
## kimeklis.K.29   K3      AY       K3-AY   11  820 7.8    8.88      28.57 0.48
## kimeklis.K.2    K1       O        K1-O  123  515 7.6   22.95      20.24 1.47
## kimeklis.K.30   K3      AY       K3-AY   11  820 7.8    8.88      28.57 0.48
## kimeklis.K.31   K3       C        K3-C    5   56 8.1    0.67       4.80 0.05
## kimeklis.K.32   K3       C        K3-C    5   56 8.1    0.67       4.80 0.05
## kimeklis.K.33   K3       C        K3-C    5   56 8.1    0.67       4.80 0.05
## kimeklis.K.34   K3       C        K3-C    5   56 8.1    0.67       4.80 0.05
## kimeklis.K.35   K3       C        K3-C    5   56 8.1    0.67       4.80 0.05
## kimeklis.K.3    K1       O        K1-O  123  515 7.6   22.95      20.24 1.47
## kimeklis.K.4    K1       O        K1-O  123  515 7.6   22.95      20.24 1.47
## kimeklis.K.56   K6      AY        K6-A  285 1110 7.7   11.70      23.81 0.58
## kimeklis.K.57   K6      AY        K6-A  285 1110 7.7   11.70      23.81 0.58
## kimeklis.K.58   K6      AY        K6-A  285 1110 7.7   11.70      23.81 0.58
## kimeklis.K.59   K6      AY        K6-A  285 1110 7.7   11.70      23.81 0.58
## kimeklis.K.5    K1       O        K1-O  123  515 7.6   22.95      20.24 1.47
## kimeklis.K.60   K6      AY        K6-A  285 1110 7.7   11.70      23.81 0.58
## kimeklis.K.6    K1      AY       K1-AY   12  212 8.0    7.32      34.50 0.78
## kimeklis.K.7    K1      AY       K1-AY   12  212 8.0    7.32      34.50 0.78
## kimeklis.K.8    K1      AY       K1-AY   12  212 8.0    7.32      34.50 0.78
## kimeklis.K.9    K1      AY       K1-AY   12  212 8.0    7.32      34.50 0.78
```

```
metadata$Description <- as.character(metadata$Description)
metadata$Description[metadata$Description == "K6-A"] <- "K6-AY"
metadata
```

```
##               Site Horizon Description P2O5  K2O  pH Organic Carbonates    N
## kimeklis.K.10   K1      AY       K1-AY   12  212 8.0    7.32      34.50 0.78
## kimeklis.K.11   K1      AC       K1-AC    6   45 8.2    0.23      33.12 0.03
## kimeklis.K.12   K1      AC       K1-AC    6   45 8.2    0.23      33.12 0.03
## kimeklis.K.13   K1      AC       K1-AC    6   45 8.2    0.23      33.12 0.03
## kimeklis.K.14   K1      AC       K1-AC    6   45 8.2    0.23      33.12 0.03
## kimeklis.K.15   K1      AC       K1-AC    6   45 8.2    0.23      33.12 0.03
## kimeklis.K.16   K2      AY       K2-AY    8  595 7.9    6.84      34.13 0.10
## kimeklis.K.17   K2      AY       K2-AY    8  595 7.9    6.84      34.13 0.10
## kimeklis.K.18   K2      AY       K2-AY    8  595 7.9    6.84      34.13 0.10
## kimeklis.K.19   K2      AY       K2-AY    8  595 7.9    6.84      34.13 0.10
## kimeklis.K.1    K1       O        K1-O  123  515 7.6   22.95      20.24 1.47
## kimeklis.K.20   K2      AY       K2-AY    8  595 7.9    6.84      34.13 0.10
## kimeklis.K.21   K2       C        K2-C    2   14 8.2    0.47      45.60 0.43
## kimeklis.K.22   K2       C        K2-C    2   14 8.2    0.47      45.60 0.43
## kimeklis.K.23   K2       C        K2-C    2   14 8.2    0.47      45.60 0.43
## kimeklis.K.24   K2       C        K2-C    2   14 8.2    0.47      45.60 0.43
## kimeklis.K.25   K2       C        K2-C    2   14 8.2    0.47      45.60 0.43
## kimeklis.K.26   K3      AY       K3-AY   11  820 7.8    8.88      28.57 0.48
## kimeklis.K.27   K3      AY       K3-AY   11  820 7.8    8.88      28.57 0.48
## kimeklis.K.28   K3      AY       K3-AY   11  820 7.8    8.88      28.57 0.48
## kimeklis.K.29   K3      AY       K3-AY   11  820 7.8    8.88      28.57 0.48
## kimeklis.K.2    K1       O        K1-O  123  515 7.6   22.95      20.24 1.47
## kimeklis.K.30   K3      AY       K3-AY   11  820 7.8    8.88      28.57 0.48
## kimeklis.K.31   K3       C        K3-C    5   56 8.1    0.67       4.80 0.05
## kimeklis.K.32   K3       C        K3-C    5   56 8.1    0.67       4.80 0.05
## kimeklis.K.33   K3       C        K3-C    5   56 8.1    0.67       4.80 0.05
## kimeklis.K.34   K3       C        K3-C    5   56 8.1    0.67       4.80 0.05
## kimeklis.K.35   K3       C        K3-C    5   56 8.1    0.67       4.80 0.05
## kimeklis.K.3    K1       O        K1-O  123  515 7.6   22.95      20.24 1.47
## kimeklis.K.4    K1       O        K1-O  123  515 7.6   22.95      20.24 1.47
## kimeklis.K.56   K6      AY       K6-AY  285 1110 7.7   11.70      23.81 0.58
## kimeklis.K.57   K6      AY       K6-AY  285 1110 7.7   11.70      23.81 0.58
## kimeklis.K.58   K6      AY       K6-AY  285 1110 7.7   11.70      23.81 0.58
## kimeklis.K.59   K6      AY       K6-AY  285 1110 7.7   11.70      23.81 0.58
## kimeklis.K.5    K1       O        K1-O  123  515 7.6   22.95      20.24 1.47
## kimeklis.K.60   K6      AY       K6-AY  285 1110 7.7   11.70      23.81 0.58
## kimeklis.K.6    K1      AY       K1-AY   12  212 8.0    7.32      34.50 0.78
## kimeklis.K.7    K1      AY       K1-AY   12  212 8.0    7.32      34.50 0.78
## kimeklis.K.8    K1      AY       K1-AY   12  212 8.0    7.32      34.50 0.78
## kimeklis.K.9    K1      AY       K1-AY   12  212 8.0    7.32      34.50 0.78
```

```
metadata$Description <- factor(metadata$Description, levels =  c("K1-O", "K1-AY", "K1-AC", "K2-AY", "K2-C", "K3-AY", "K3-C", "K6-AY"))
metadata <- metadata[order(metadata$Description, decreasing = FALSE),]
#colnames(metadata) <- 
colnames(metadata) <- c("Site", "Horizon", "Description" ,"P2O5", "K2O","pH","TOC","C", "N")
sample_data(ps) <- metadata
```

### Richness/depth correlation.

```
library(phyloseq)
library(ggplot2)
plot_rich_reads_samlenames_lm <- function(physeq){
  rish <- estimate_richness(physeq, measures = "Observed")
  reads.sum <- as.data.frame(sample_sums(physeq))
  reads.summary <- cbind(rish, reads.sum)
  colnames(reads.summary) <- c("otus","reads")
  reads.summary["Description"] <-unlist(purrr::map(stringr::str_split(rownames(physeq@sam_data), "K.", 2), function(x) x[[2]]))
  reads.summary["Repeats"] <- physeq@sam_data$Description
  reads.summary["Plant"] <- physeq@sam_data$Plant
  library(ggrepel)
  require(ggforce)
  p1 <- ggplot(data=reads.summary) + geom_point(aes(y=otus, x=log2(reads), color=Repeats),size=3) + geom_text_repel(aes(y=otus, x=log2(reads), label=paste0(Repeats, "_", Description))) + theme_bw()+
    geom_smooth(aes(y=otus, x=log2(reads), fill=Repeats, color=Repeats),method=lm, se=FALSE, ymin = 1) + scale_x_continuous(sec.axis = sec_axis(sec.axis ~ 2**.)) 
  # geom_mark_ellipse(aes(y = otus, x=reads, group = Repeats, label = Repeats, color = Repeats), label.fontsize = 10, label.buffer = unit(2, "mm"), label.minwidth = unit(5, "mm"),con.cap = unit(0.1, "mm"))
  
  return(p1)
}

plot_rich_reads_samlenames_lm(ps)
```

```
ps.f <-  subset_samples(ps, sample_names(ps) != 'kimeklis.K.20')
ps.f <-  subset_samples(ps.f, sample_names(ps.f) != 'kimeklis.K.22')
ps.f <-  subset_samples(ps.f, sample_names(ps.f) != 'kimeklis.K.23')
ps.f <- prune_taxa(taxa_sums(ps.f) > 0, ps.f)
plot_rich_reads_samlenames_lm(ps.f)
```

### Alpha diversity violins.

```
library(ggpubr)
alpha.custom <- function(ps.f, arrga = "PD"){
  require(picante)
  pd <- pd(ps.f@otu_table@.Data, ps.f@phy_tree)
  
  pd1 <- estimate_richness(ps.f, measures=c("Observed", "InvSimpson", "Shannon"))
  pd <- cbind(pd,ps.f@sam_data, pd1 )
  
  bmi <- levels(as.factor(pd$Description))
  bmi.pairs <- combn(seq_along(bmi), 2, simplify = FALSE, FUN = function(i)bmi[i])
  p1 <- ggviolin(pd, x = "Description", y = arrga,
                 add = "boxplot", fill = "Description") + stat_compare_means(comparisons = bmi.pairs, method = "wilcox.test") +
    scale_x_discrete(limits = c("K1-O", "K1-AY", "K1-AC", "K2-AY", "K2-C", "K3-AY", "K3-C", "K6-AY")) + theme(axis.title.x = element_blank(), legend.title = element_blank())
  return(p1)
}

p.alpha.Cdt.oo <- alpha.custom(ps.f, arrga = "Observed")
p.alpha.Cdt.sh <- alpha.custom(ps.f, arrga = "PD")
p.alpha.Cdt.is <- alpha.custom(ps.f, arrga = "Shannon")
p.alpha.Cdt.pd <- alpha.custom(ps.f, arrga = "InvSimpson")


p1 <- ggarrange(p.alpha.Cdt.oo, p.alpha.Cdt.sh,p.alpha.Cdt.is, p.alpha.Cdt.pd , ncol = 4 ,label.x = 0.105, nrow = 1, common.legend = TRUE)
p1
```

### Heatmaps with relative abundance by ampvis2 package

```
phyloseq_to_amp <- function(ps){
    require(ampvis2)
    require(tibble)
    require(phyloseq)
    colnames(ps@tax_table@.Data) <- c("Kingdom", "Phylum", "Class", "Order", "Family", "Genus", "Species")
    OTU1 = as(otu_table(ps), "matrix")
    OTU1 <- t(OTU1)
    OTUdf = as.data.frame(OTU1)
    taxa.ps <- as(tax_table(ps), "matrix")
    taxa.df = as.data.frame(taxa.ps)
    my_otu_table <- merge(OTUdf, taxa.df, by=0)
    my_otu_table <- column_to_rownames(my_otu_table, var="Row.names")

    my_metadata <- as_tibble(sample_data(ps), rownames=NA)
    my_metadata <- rownames_to_column(my_metadata,var = "SampleID")
    my_tree <- phy_tree(ps)
    amp.ps <- amp_load(otutable = my_otu_table, metadata = my_metadata, tree = my_tree)
    return(amp.ps)
}
amp <- phyloseq_to_amp(ps.f)
```

```
## Warning in class(x) <- c(setdiff(subclass, tibble_class), tibble_class): Setting class(x) to multiple strings ("tbl_df", "tbl", ...); result will no longer be an S4 object
```

```
amp_heatmap(amp, group_by = "Description",tax_add = "Phylum", tax_show = 40, tax_aggregate = "Family", order_x_by = c("K1-O", "K1-AY", "K1-AC", "K2-AY", "K2-C", "K3-AY", "K3-C", "K6-AY")) + theme_bw() + theme(text = element_text(size=15), legend.position = "none")
```

```
amp_heatmap(amp, group_by = "Description",tax_show = 20, tax_aggregate = "Phylum") + theme_bw() + theme(text = element_text(size=15), legend.position = "none")
```

```
## Warning: Transformation introduced infinite values in discrete y-axis
```

### Beta-diversity plots

```
beta_custom_norm_NMDS_elli <- function(ps, seed = 7888, normtype="vst", color="Description"){
  require(phyloseq)
  require(ggplot2)
  require(ggpubr)
  require(DESeq2)
  library(ggforce)
  
  
  # beta_NMDS <- function(){
  #normalisation. unifrac - rarefaction; wunifrac,bray - varstab
  
  diagdds = phyloseq_to_deseq2(ps, ~ Description)                  
  diagdds = estimateSizeFactors(diagdds, type="poscounts")
  diagdds = estimateDispersions(diagdds, fitType = "local") 
  if (normtype =="vst")
    pst <- varianceStabilizingTransformation(diagdds)
  if (normtype =="log") 
    pst <- rlogTransformation(diagdds)
  
  pst.dimmed <- t(as.matrix(assay(pst))) 
  pst.dimmed[pst.dimmed < 0.0] <- 0.0
  ps.varstab <- ps
  otu_table(ps.varstab) <- otu_table(pst.dimmed, taxa_are_rows = FALSE) 
  
  ps.rand <- rarefy_even_depth(ps, rngseed = seed)
  
  #beta and ordination
  
  ordination.b <- ordinate(ps.varstab, "NMDS", "bray")
  ordination.u <- ordinate(ps.rand, "NMDS", "unifrac")
  ordination.w <- ordinate(ps.varstab, "NMDS", "wunifrac")
  
  #plotting
  p1 = plot_ordination(ps, ordination.b, type="sample", color="Description", title="NMDS - Bray-Curtis", 
                       axes = c(1,2) ) + theme_bw() + theme(text = element_text(size = 10)) + geom_point(size = 3) +
    geom_mark_ellipse(aes(group = Description, label = Description), label.fontsize = 10, label.buffer = unit(2, "mm"), label.minwidth = unit(5, "mm"),con.cap = unit(0.1, "mm"))
  
  p2 = plot_ordination(ps, ordination.u, type="sample", color="Description", title="NMDS - UniFrac", 
                       axes = c(1,2) ) + theme_bw() + theme(text = element_text(size = 10)) + geom_point(size = 3) +
    geom_mark_ellipse(aes(group = Description, label = Description), label.fontsize = 10, label.buffer = unit(2, "mm"), label.minwidth = unit(5, "mm"),con.cap = unit(0.1, "mm"))
  
  p3 = plot_ordination(ps, ordination.w, type="sample", color="Description", title="NMDS - Weighted UniFrac", 
                       axes = c(1,2) ) + theme_bw() + theme(text = element_text(size = 10)) + geom_point(size = 3) + 
    geom_mark_ellipse(aes(group = Description, label = Description), label.fontsize = 10, label.buffer = unit(2, "mm"), label.minwidth = unit(5, "mm"),con.cap = unit(0.1, "mm"))
  
  #merge by ggpubr
  
  p.all <- ggarrange(p1, p2, p3, ncol = 3 , nrow = 1, common.legend = TRUE, legend = "none", font.label = list(size = 12, face = "bold", color ="black"))
  
  return(p.all)
}

p.beta <- beta_custom_norm_NMDS_elli(ps.f)
p.beta
```

### PERMANOVA All factors

```
permanova.forloop.noNestedPlant <- function(factor){
  require(vegan)
  require(phyloseq)
  dist <- phyloseq::distance(physeq, "bray")
  metadata <- as(sample_data(physeq@sam_data), "data.frame")
  ad <- adonis2(as.formula(paste( "dist ~ ", factor)), data = metadata)
  ad <- cbind2(ad[1,][3], ad[1,][5])
  return(ad)
}

physeq <- ps.f
col.physeq <- colnames(physeq@sam_data)
col.physeq <- col.physeq[ col.physeq != "Description"]

permanova.forloop.pos.noPlant <- purrr::possibly(permanova.forloop.noNestedPlant, otherwise = NA)
out <- purrr::map(col.physeq, permanova.forloop.pos.noPlant)
out.d.noPlant <- do.call("rbind", out)
out.d.noPlant[order(out.d.noPlant$R2, decreasing = TRUE),]
```

```
##                 R2 Pr(>F)
## Site    0.49421618  0.001
## Horizon 0.43021747  0.001
## N       0.18573955  0.001
## TOC     0.17705733  0.001
## pH      0.15795176  0.001
## K2O     0.14989736  0.001
## P2O5    0.11987081  0.001
## C       0.04737008  0.110
```

### PERMANOVA are nested by horizon

```
permanova.forloop.noNestedPlant <- function(factor){
  require(vegan)
  require(phyloseq)
  dist <- phyloseq::distance(physeq, "bray")
  metadata <- as(sample_data(physeq@sam_data), "data.frame")
  ad <- adonis2(as.formula(paste( "dist ~ Horizon/", factor)), data = metadata)
  ad <- cbind2(ad[2,][3], ad[2,][5])
  return(ad)
}

physeq <- ps.f
col.physeq <- colnames(physeq@sam_data)
col.physeq <- col.physeq[ col.physeq != c("Description")]

permanova.forloop.pos.noPlant <- purrr::possibly(permanova.forloop.noNestedPlant, otherwise = NA)
out <- purrr::map(col.physeq, permanova.forloop.pos.noPlant)
out.d.noPlant <- do.call("rbind", out)
out.d.noPlant[order(out.d.noPlant$R2, decreasing = TRUE),]
```

```
##                     R2 Pr(>F)
## Residual     0.5697825     NA
## Horizon:Site 0.3321851  0.001
## Horizon:N    0.1637269  0.001
## Horizon:K2O  0.1478728  0.001
## Horizon:pH   0.1377684  0.001
## Horizon:P2O5 0.1126650  0.001
## Horizon:C    0.1097756  0.001
## Horizon:TOC  0.1051342  0.001
```

## CCA

```
library(phyloseq)             
library(vegan)

ps.merged <- merge_samples(ps.f, "Description")
physeq <- ps.merged

veganifyOTU <- function(physeq){
  require(phyloseq)
  if(taxa_are_rows(physeq)){physeq <- t(physeq)}
  return(as(otu_table(physeq), "matrix"))
}


otus.ps.vegan <- veganifyOTU(physeq)

metadata <- as(sample_data(physeq), "data.frame")

vare.cca <- vegan::cca(otus.ps.vegan ~  N + TOC + C + pH + K2O + P2O5, data=metadata)


kate.ggcca.sites <- function(vare.cca){

require(tidyverse)
biplot <- as.data.frame(vare.cca$CCA$biplot)
wa <- as.data.frame(vare.cca$CCA$wa)

biplot <- rownames_to_column(biplot, "Label") %>% 
  add_column(Score = rep("biplot", length(rownames(biplot))))
wa <- rownames_to_column(wa, "Label") %>% 
  add_column(Score = rep("sites", length(rownames(wa))))
fdat_amazing <- rbind(biplot, wa)

 
p <- ggplot(fdat_amazing %>% filter(Score %in% c("sites","biplot"))) + 
  geom_point(data = fdat_amazing %>% dplyr::filter(Score == "sites"), mapping = aes(x=CCA1, y=CCA2, colour = factor(Score))) + 
  geom_segment(data = fdat_amazing %>% dplyr::filter(Score == "biplot"), aes(x = 0, xend = CCA1, y = 0, yend = CCA2), alpha=0.8, color = "red",arrow = arrow(angle = 3)) +
  geom_text_repel(aes(x=CCA1, y=CCA2, label= Label),size=4) + 
  theme(legend.position = "none", panel.background = element_rect(fill = "white", colour = "grey50"))
  return(p)
}
kate.ggcca.sites(vare.cca)
```

### CCA statistics

```
anova(vare.cca)
```

```
## Permutation test for cca under reduced model
## Permutation: free
## Number of permutations: 999
## 
## Model: cca(formula = otus.ps.vegan ~ N + TOC + C + pH + K2O + P2O5, data = metadata)
##          Df ChiSquare      F Pr(>F)
## Model     6   1.46588 1.5235  0.133
## Residual  1   0.16036
```

#### axis

```
anova(vare.cca, by="terms")
```

```
## Permutation test for cca under reduced model
## Terms added sequentially (first to last)
## Permutation: free
## Number of permutations: 999
## 
## Model: cca(formula = otus.ps.vegan ~ N + TOC + C + pH + K2O + P2O5, data = metadata)
##          Df ChiSquare      F Pr(>F)  
## N         1   0.35556 2.2172  0.055 .
## TOC       1   0.21456 1.3379  0.247  
## C         1   0.14223 0.8869  0.560  
## pH        1   0.26525 1.6541  0.154  
## K2O       1   0.30221 1.8846  0.090 .
## P2O5      1   0.18607 1.1603  0.353  
## Residual  1   0.16036                
## ---
## Signif. codes:  0 '***' 0.001 '**' 0.01 '*' 0.05 '.' 0.1 ' ' 1
```

#### multicolleniarity

```
vif.cca(vare.cca)
```

```
##          N        TOC          C         pH        K2O       P2O5 
##  12.586457 309.966091  10.443870 688.361650 171.632543   7.990314
```

#### margins

```
anova(vare.cca, by="mar")
```

```
## Permutation test for cca under reduced model
## Marginal effects of terms
## Permutation: free
## Number of permutations: 999
## 
## Model: cca(formula = otus.ps.vegan ~ N + TOC + C + pH + K2O + P2O5, data = metadata)
##          Df ChiSquare      F Pr(>F)
## N         1   0.10590 0.6604  0.771
## TOC       1   0.25883 1.6141  0.168
## C         1   0.18453 1.1507  0.337
## pH        1   0.25116 1.5662  0.174
## K2O       1   0.21595 1.3466  0.247
## P2O5      1   0.18607 1.1603  0.341
## Residual  1   0.16036
```

#### Split dataset by sites AY 1/3

```
ps.f <- subset_taxa(ps.f, !is.na(phylum))

ps.ay <- prune_samples(sample_data(ps.f)$Horizon %in% c("AY"),ps.f)
ps.ay  <- prune_taxa(taxa_sums(ps.ay) > 0, ps.ay)

ps.ay.w2 <- prune_samples(sample_data(ps.ay)$Site != c("K2"), ps.ay)
ps.ay.w2 <- prune_taxa(taxa_sums(ps.ay.w2) > 0, ps.ay.w2)

ps.c <- prune_samples(sample_data(ps.f)$Horizon %in% c("C", "AC"),ps.f)
ps.c  <- prune_taxa(taxa_sums(ps.c) > 0, ps.c)


ps.c.w2 <- prune_samples(sample_data(ps.c)$Site != c("K2"), ps.c)
ps.c.w2 <- prune_taxa(taxa_sums(ps.c.w2) > 0, ps.c.w2)
```

#### Improve on des function - add a required factor(with as.formula function)

```
des_w_simper <- function(ps, factor){
  require(DESeq2)
  require(vegan)
  require(tibble)
  require(phyloseq)
  
  diagdds = phyloseq_to_deseq2(ps, as.formula(paste( "~", factor)))                  
  diagdds = estimateSizeFactors(diagdds, type="poscounts")
  diagdds = estimateDispersions(diagdds, fitType = "local") 
  diagdds = DESeq(diagdds)
  samp <-sample_data(ps)
  aggdata <- t(aggregate.data.frame(as.data.frame(as.data.frame(t(diagdds@assays@data$mu))), by=list(samp[[factor]]), median))
  colnames(aggdata) <- aggdata[1,]
  aggdata <- aggdata[-1,]
  res = results(diagdds)
  res.df <- as.data.frame(res)
  nice <- cbind(res.df, as.data.frame(ps@tax_table@.Data[rownames(res.df),]), as.data.frame(aggdata)[rownames(res.df),])
} 

des.ay <- des_w_simper(ps.ay.w2, "Site")
des.c <- des_w_simper(ps.c.w2, "Site")
```

#### filtration ASVs in the DESeq2 result tables by abundanse and change confidence level

```
filter.des <- function(des, alpha=0.1, baseMean = 15){
  des <- des[(des$padj < alpha), ]
  des <- des[(des$baseMean > baseMean),]
  #des <- des[(des$log2FoldChange > 0),]
  des <-  des[(is.na(des$padj) != TRUE),]
  return(des)
}
alpha.c <- 0.05
baseMean.c <- 60
des.c.filt <- filter.des(des.c, alpha = alpha.c, baseMean = baseMean.c)
len.c <- length(rownames(des.c.filt))
paste0("length des.c after filtation with param: alpha - ", alpha.c, ", baseMean - ", baseMean.c, " ==> ", len.c)
```

```
## [1] "length des.c after filtation with param: alpha - 0.05, baseMean - 60 ==> 45"
```

```
alpha.ay <- 0.05
baseMean.ay <- 60
des.ay.filt <- filter.des(des.ay, alpha = alpha.ay, baseMean = baseMean.ay)
len.ay <- length(rownames(des.ay.filt))
paste0("length des.ay  after filtation with param: alpha - ", alpha.ay, ", baseMean - ", baseMean.ay, " ==> ", len.ay)
```

```
## [1] "length des.ay  after filtation with param: alpha - 0.05, baseMean - 60 ==> 26"
```

#### Tree for soil changed taxa

```
list.both <- c(rownames(des.c.filt), rownames(des.ay.filt)) 
ps.changed <- prune_taxa(list.both, ps.f)
phyloseq <- ps.changed
library(ggtree)
library(tidyverse)
tree <- phyloseq@phy_tree
taxa.pruned <- as.data.frame(phyloseq@tax_table@.Data)
taxa.pruned <- taxa.pruned %>%  mutate_all(as.character)
old.tips <- tree$tip.label
matches <- regmatches(tree$tip.label, gregexpr("[[:digit:]]+", tree$tip.label))
taxa.pruned$number <- as.numeric(unlist(matches))
taxa.pruned$taxa <- ifelse(is.na(taxa.pruned$genus), taxa.pruned$family, taxa.pruned$genus)
taxa.pruned[taxa.pruned == "Burkholderia-Caballeronia-Paraburkholderia"] <- "Burkholderia"
taxa.pruned[taxa.pruned == "Allorhizobium-Neorhizobium-Pararhizobium-Rhizobium"] <- "Pararhizobium"
taxa.pruned$taxa2 <- ifelse(is.na(taxa.pruned$species), with(taxa.pruned, paste0(taxa)), with(taxa.pruned, paste0(taxa, " ", species )))
taxa.pruned$taxa3 <- ifelse(taxa.pruned$phylum == "Proteobacteria", with(taxa.pruned, paste0(taxa.pruned$number, ".", taxa2, " // ", class)), with(taxa.pruned, paste0(taxa.pruned$number, ".", taxa2, " // ", phylum)))
tree$tip.label <- taxa.pruned$taxa3
p <- ggtree(tree, ladderize = F) + geom_tiplab(mapping = aes(), align=TRUE, linesize=.5) + xlim(NA, 4)
```

```
## Warning: `data_frame()` is deprecated as of tibble 1.1.0.
## Please use `tibble()` instead.
## This warning is displayed once every 8 hours.
## Call `lifecycle::last_warnings()` to see where this warning was generated.
```

#### Add log2FoldChange for tree. Red - prevalence in CZ, blue - in SP.

```
t.ay <-   as_tibble(des.ay, rownames = "id") %>% 
  filter(id %in% phyloseq@phy_tree$tip.label) %>% 
  select(id, log2FoldChange) %>%  
  rename(ay = log2FoldChange)

t.c <- as_tibble(des.c, rownames = "id") %>% 
  filter(id %in% phyloseq@phy_tree$tip.label) %>% 
  select(id, log2FoldChange) %>%  
  rename(c = log2FoldChange)

t.all <- full_join(t.ay, t.c)

names.d <-  cbind(long = tree$tip.label, short = old.tips) %>% data.frame(row.names = "short")
t.names <- as_tibble(names.d, rownames = "id")
d.all.tips <- full_join(t.names, t.all) %>%  select(-c(id)) %>% 
  as.data.frame() %>% column_to_rownames("long")

p2 <- gheatmap(p, d.all.tips, offset=2, width=0.8)  + scale_colour_gradientn(colours = c("blue", "white", "red"),na.value = "grey50", guide = "colourbar",aesthetics = "fill") 
p2 <- p2 + labs(fill = "L2FC K1/K3")
```

```
otus.ay.rel <- t(apply(otu_table(ps.ay), 1, function(x) x / sum(x)))
otus.c.rel <- t(apply(otu_table(ps.c), 1, function(x) x / sum(x)))
# for ay
as_tibble(t(otus.ay.rel ), rownames = "id" ) %>% 
  filter(id %in% phyloseq@phy_tree$tip.label) %>%
  select(id) -> id.ay
as_tibble(t(otus.ay.rel), rownames = "id" ) %>% 
  filter(id %in% phyloseq@phy_tree$tip.label) %>%
  select_if(is.numeric) %>% 
  replace(is.na(.), 0) %>% 
  mutate(ayMean=rowMeans(.)) %>% 
  select(ayMean) -> res.ay
res.ayMean.rel <- cbind2(id.ay,res.ay)

# for c
as_tibble(t(otus.c.rel), rownames = "id" ) %>% 
  filter(id %in% phyloseq@phy_tree$tip.label) %>%
  select(id) -> id.c
as_tibble(t(otus.c.rel), rownames = "id" ) %>% 
  filter(id %in% phyloseq@phy_tree$tip.label) %>%
  select_if(is.numeric) %>% 
  replace(is.na(.), 0) %>% 
  mutate(cMean=rowMeans(.)) %>% 
  select(cMean) -> res.c
res.cMean.rel <- cbind2(id.c, res.c)


all.resMean.rel <- full_join(res.ayMean.rel, res.cMean.rel)
all.resMean.rel %>% replace(is.na(.), 0) -> all.resMean.rel
all.resMean.rel$ayMean <- 0 - all.resMean.rel$ayMean
all.resMean.rel.melted <- reshape2::melt(all.resMean.rel, id=c("id"))

library(reshape2)
library(ggstance)
all.resMean.rel.melted <- melt(all.resMean.rel, id=c("id"))
first.tree <- phyloseq@phy_tree
ids.tree <-  data.frame(id = first.tree$tip.label, id.taxa = tree$tip.label)
some.join <- full_join(ids.tree, all.resMean.rel.melted)
```

```
## Warning: Column `id` joining factor and character vector, coercing into character vector
```

```
some.join <- subset(some.join, select = -c(id))
some.join["numbers"] <-  ifelse(some.join$value == 0, NA, abs(some.join$value))
some.join$value <- some.join$value*100
some.join["position"] <-  ifelse(some.join$value >= 0, some.join$value + 0.5, some.join$value - 0.5)
p4 <- facet_plot(p2, panel = "AY / C", data = some.join,  geom = geom_barh, 
                 mapping = aes(x = value, label=value), color = "salmon", fill = "white",
                 stat='identity')
```

```
## Warning: Ignoring unknown aesthetics: label
```

```
p4 <- facet_plot(p4, geom=geom_text , data = some.join, mapping=aes(x=position,label=round(numbers*100, digits = 2)), panel = "AY / C")

library(gtable)
library(grid)
gt <-  ggplot_gtable(ggplot_build(p4))
gt$widths[7] <-  0.5*gt$widths[7] # in this case it was colmun 7 - reduce the width by a half
plot(gt) # plot with grid draw
```

### Uncorrect p-value cheking on the phyla level by DESeq2

The first 20 most abundant phyla

#### Between AY and C for 2 and 3 sites

```
des_w_simper <- function(ps, factor){
  require(DESeq2)
  require(vegan)
  require(tibble)
  require(phyloseq)
  
  diagdds = phyloseq_to_deseq2(ps, as.formula(paste( "~", factor)))                  
  diagdds = estimateSizeFactors(diagdds, type="poscounts")
  diagdds = estimateDispersions(diagdds, fitType = "local") 
  diagdds = DESeq(diagdds)
  samp <-sample_data(ps)
  aggdata <- t(aggregate.data.frame(as.data.frame(as.data.frame(t(diagdds@assays@data$mu))), by=list(samp[[factor]]), median))
  colnames(aggdata) <- aggdata[1,]
  aggdata <- aggdata[-1,]
  res = results(diagdds)
  res.df <- as.data.frame(res)
  nice <- cbind(res.df, as.data.frame(ps@tax_table@.Data[rownames(res.df),]), as.data.frame(aggdata)[rownames(res.df),])
} 

ps.23 <- prune_samples(sample_data(ps.f)$Site %in% c("K2", "K3"), ps.f)
ps.23  <- prune_taxa(taxa_sums(ps.23) > 0, ps.23)

ps.23.phylum <- tax_glom(ps.23,  "phylum")


des.23.phylum <- des_w_simper(ps.23.phylum, "Horizon")
des.23.little <- des.23.phylum[,c("phylum", "baseMean" , "log2FoldChange", "padj", "AY", "C")]
des.23.little[order(des.23.little$baseMean, decreasing = TRUE),]
```

```
##                       phylum     baseMean log2FoldChange         padj        AY         C
## Seq4          Actinobacteria 6301.5255960    -0.05491843 6.294639e-01  8039.421  5219.175
## Seq15         Proteobacteria 5209.6795710    -0.41950555 1.918988e-04  7410.866  3736.752
## Seq19          Acidobacteria 3279.0575887     0.25727757 1.406927e-02  3764.159  3034.069
## Seq7           Bacteroidetes 3053.3218936    -0.97846494 1.368254e-08  4982.329  1705.274
## Seq1          Thaumarchaeota 1826.6375638     0.94448324 7.659076e-05  1595.531  2070.772
## Seq5         Verrucomicrobia 1695.6316976     0.72871180 6.394829e-03  1623.370  1814.223
## Seq3              Firmicutes 1649.1663526     0.76682627 6.553081e-02   1554.13   1783.34
## Seq279        Planctomycetes 1375.9479832    -0.14256017 7.743462e-01  1804.466  1102.410
## Seq250           Chloroflexi  499.2900010    -0.44428638 1.182226e-02  715.1036  354.4334
## Seq13          Cyanobacteria  397.2302571    -2.98485111 1.500616e-07 845.67138  72.04122
## Seq140      Gemmatimonadetes  320.2904724     0.03327555 9.116064e-01  396.9901  273.9717
## Seq51      Entotheonellaeota  301.6643230     0.34629107 2.416710e-01  335.2299  287.4061
## Seq1004      Patescibacteria  183.1121783     0.60765956 8.372335e-04  184.7009  189.8025
## Seq104          Rokubacteria   75.1990384     2.58469929 2.170211e-28  28.14306 113.85508
## Seq750                   FBP   66.8784249    -2.29237238 5.832548e-11 134.04283  18.45355
## Seq3158      Armatimonadetes   51.1700534    -0.90210189 2.222247e-06  81.49297  29.40827
## Seq161           Nitrospirae   35.1831905    -0.05203801 9.116064e-01  44.84191  29.16945
## Seq1603        Elusimicrobia   15.7648539    -0.15889300 8.019380e-01  20.96892  12.66641
## Seq3581         Dependentiae   12.9131278     3.22138652 2.536225e-12  3.302095 20.769813
## Seq6494           Chlamydiae    8.9109392     0.34416406 6.284375e-01 10.072363  8.622718
## Seq6371                 BRC1    6.1118797    -0.82385602 1.258233e-01  9.729961  3.706934
## Seq4706        Fibrobacteres    4.1991357    -1.04857792 2.416710e-01  7.055137  2.300177
## Seq9229      Nanoarchaeaeota    3.1238753     1.05955962 4.987620e-01  2.630226  3.697101
## Seq4958      Hydrogenedentes    2.7344146    -1.14953387 2.627495e-01  4.615518  1.403090
## Seq8424      Latescibacteria    2.5641341     3.16455815 2.471961e-04 0.6945295 4.1997819
## Seq8109  Deinococcus-Thermus    2.0429016    -3.71925103 8.238141e-03 4.5628780 0.2336371
## Seq5934        Euryarchaeota    1.9267378     1.39428326 2.323379e-01  1.359727  2.410360
## Seq7482     Omnitrophicaeota    1.6258513    -0.80498818 4.987620e-01 2.5111852 0.9693091
## Seq5493                WPS-2    1.0430037    -0.67655574 6.284375e-01 1.6026762 0.6762252
## Seq8343   Kiritimatiellaeota    0.3087374     2.15335470 6.284375e-01 0.2005017 0.6015221
## Seq10814                 WS2    0.1387764     0.14781758 9.574199e-01 0.3127043 0.2336364
## Seq8586                GAL15    0.1168035     0.78900475 8.786140e-01 0.2005009 0.2336359
```

#### For site 2

```
ps.23 <- prune_samples(sample_data(ps.f)$Site %in% c("K2"), ps.f)
ps.23  <- prune_taxa(taxa_sums(ps.23) > 0, ps.23)

ps.23.phylum <- tax_glom(ps.23,  "phylum")


des.23.phylum <- des_w_simper(ps.23.phylum, "Horizon")
des.23.little <- des.23.phylum[,c("phylum", "baseMean" , "log2FoldChange", "padj", "AY", "C")]
des.23.little[order(des.23.little$baseMean, decreasing = TRUE),]
```

```
##                       phylum     baseMean log2FoldChange              padj        AY         C
## Seq4          Actinobacteria 6457.5907320    -0.21845165 0.401211125447261  7958.172  5597.208
## Seq15         Proteobacteria 5033.3344239    -0.33952153 0.095802174023108  6405.788  4142.715
## Seq7           Bacteroidetes 3307.7219336    -0.75106977 0.000147368928684  4638.141  2255.112
## Seq19          Acidobacteria 3241.6096078    -0.01450181 0.914243686737176  3770.754  3054.790
## Seq1          Thaumarchaeota 2067.3495622     0.91118057 0.001357623531849  1738.177  2674.886
## Seq3              Firmicutes 1309.6522692     1.54864500 0.000000482711331  830.7717 1988.7900
## Seq279        Planctomycetes 1268.4025696    -0.74555480 0.051335235246781 1777.0267  867.3177
## Seq5         Verrucomicrobia 1189.2145011     0.17280312 0.535094208211581  1306.765  1205.410
## Seq250           Chloroflexi  473.1249912    -0.19121037 0.446948685049592  579.6650  415.4662
## Seq51      Entotheonellaeota  364.5301562    -0.26471555 0.428405026666922  454.4883  309.5666
## Seq268      Gemmatimonadetes  297.8677676     0.39154859 0.428405026666922  304.0660  326.4031
## Seq1866      Patescibacteria  175.8717753     0.50339104 0.186349332370725  173.6635  201.4480
## Seq73          Cyanobacteria  168.6885307    -1.87808327 0.000052584805316 283.54993  63.12317
## Seq750                   FBP   85.0924774    -2.76767654 0.000000002597082  156.0842   18.7553
## Seq104          Rokubacteria   55.9420140     2.30656779 0.000000004833719  24.10396  97.57827
## Seq3160      Armatimonadetes   52.9111173    -0.94727095 0.008312454005609  76.70889  32.55423
## Seq161           Nitrospirae   50.6023724     0.13834596 0.818795148078112  55.84219  50.29529
## Seq1603        Elusimicrobia   21.8750909     0.70126364 0.224972148493191  20.22433  26.90879
## Seq4159         Dependentiae    9.4050226     2.41666879 0.002927743007979  3.790355 16.561067
## Seq6494           Chlamydiae    8.7204203     0.66191279 0.541080898792165  8.171276 10.579476
## Seq7248                 BRC1    7.6383919    -0.65733373 0.528535264363214 10.738117  5.571467
## Seq5076      Hydrogenedentes    3.1791168    -2.78603030 0.171903450527085 5.8262325 0.6912382
## Seq8424      Latescibacteria    3.0797052     3.94283677 0.027488621286452 0.5115068 6.4369798
## Seq4706        Fibrobacteres    2.7477118     0.19112318 0.914243686737176  2.956795  2.762318
## Seq8109  Deinococcus-Thermus    2.4813577    -3.31485302 0.178838308631638 4.5905733 0.3774986
## Seq7482     Omnitrophicaeota    1.3805689     0.41276713 0.914243686737176  1.365882  1.487946
## Seq6204                WPS-2    1.3649896    -0.76924023 0.813958231489664 1.8789975 0.9021535
## Seq8665      Nanoarchaeaeota    0.8279526    -2.86503431 0.446948685049592 1.7224456 0.1934652
## Seq5934        Euryarchaeota    0.6004933     3.08432039 0.446948685049592 0.1970581 1.3676840
## Seq10814                 WS2    0.1014093    -0.45861083 0.914243686737176 0.3248903 0.1934638
```

#### For site 3

```
ps.23 <- prune_samples(sample_data(ps.f)$Site %in% c("K3"), ps.f)
ps.23  <- prune_taxa(taxa_sums(ps.23) > 0, ps.23)

ps.23.phylum <- tax_glom(ps.23,  "phylum")

des.23.phylum <- des_w_simper(ps.23.phylum, "Horizon")
des.23.little <- des.23.phylum[,c("phylum", "baseMean" , "log2FoldChange", "padj", "AY", "C")]
des.23.little[order(des.23.little$baseMean, decreasing = TRUE),]
```

```
##                       phylum     baseMean log2FoldChange         padj         AY          C
## Seq4          Actinobacteria 6185.6502430    0.008693494 9.315222e-01   8007.530   5035.367
## Seq10         Proteobacteria 5339.0233477   -0.531675056 3.866285e-03   8196.419   3543.966
## Seq19          Acidobacteria 3309.3008901    0.380819566 6.434115e-02   3732.947   3038.124
## Seq7           Bacteroidetes 2900.8424021   -1.194714731 1.159587e-05   5243.118   1431.727
## Seq5         Verrucomicrobia 2037.8713402    0.863739659 1.801414e-03   1876.703   2134.630
## Seq3              Firmicutes 1856.5498270    0.349058044 5.439981e-01   2120.441   1688.179
## Seq1          Thaumarchaeota 1664.0786448    0.929744745 1.492534e-02   1487.666   1771.340
## Seq246        Planctomycetes 1445.7245537    0.129570160 8.718199e-01   1792.654   1225.791
## Seq13          Cyanobacteria  572.0021002   -3.463596901 4.208798e-07 1362.06081   77.17302
## Seq292           Chloroflexi  516.6793296   -0.669025215 9.921645e-03   823.5642   323.7549
## Seq140      Gemmatimonadetes  334.3865911   -0.264222062 4.497756e-01   473.9220   246.6515
## Seq108     Entotheonellaeota  258.4893842    0.948097032 8.776043e-06   229.1352   276.3204
## Seq1004      Patescibacteria  187.8784224    0.630771829 2.190855e-02   191.6062   185.4413
## Seq104          Rokubacteria   87.5172312    2.633264761 6.390600e-17   31.57221  122.43686
## Seq750                   FBP   54.7096622   -1.922942579 5.890294e-05  112.18044   18.49138
## Seq3158      Armatimonadetes   50.2568895   -0.935735771 7.171999e-03   85.41579   27.91060
## Seq161           Nitrospirae   24.8631209   -0.285934851 5.819428e-01   35.66735   18.28569
## Seq3581         Dependentiae   15.2473107    3.672060689 2.247749e-07   2.898612  23.094367
## Seq1603        Elusimicrobia   11.5354525   -1.311676347 2.413162e-02  21.483124   5.409524
## Seq6310           Chlamydiae    9.1231554    0.066173719 9.315222e-01  11.704290   7.659156
## Seq4932        Fibrobacteres    5.1193518   -1.624619676 8.077675e-02  10.326597   2.093214
## Seq6371                 BRC1    5.0834252   -0.996425335 2.796736e-01   8.681804   2.720017
## Seq9229      Nanoarchaeaeota    4.6824891    1.419821798 2.760801e-01   3.372482   5.639935
## Seq9757        Euryarchaeota    2.8397091    0.926778414 4.652194e-01   2.482343   2.949616
## Seq4958      Hydrogenedentes    2.3820225   -0.215147105 9.315222e-01   3.326578   1.791214
## Seq10115     Latescibacteria    2.1560358    2.485563374 6.434115e-02  0.8537863  2.9887796
## Seq7482     Omnitrophicaeota    1.8346567   -1.605887061 2.664342e-01  3.5638941  0.7318468
## Seq8109  Deinococcus-Thermus    1.6859274   -3.934702067 7.696121e-02  4.3255232  0.1768035
## Seq5493                WPS-2    0.8692379   -0.764473757 7.604374e-01  1.4776707  0.5437055
## Seq8343   Kiritimatiellaeota    0.5248134    2.803573791 4.652194e-01  0.2016694  0.8800663
## Seq8586                GAL15    0.1973655    1.065177519 8.718199e-01  0.2016687  0.2637573
## Seq10814                 WS2    0.1619173    0.488108151 9.315222e-01  0.3008532  0.2637578
```

```
ps.f <- subset_taxa(ps.f, !is.na(phylum))

ps.ay <- prune_samples(sample_data(ps.f)$Horizon %in% c("AY"),ps.f)
ps.ay  <- prune_taxa(taxa_sums(ps.ay) > 0, ps.ay)

ps.ay.w2 <- prune_samples(sample_data(ps.ay)$Site != c("K2"), ps.ay)
ps.ay.w2 <- prune_taxa(taxa_sums(ps.ay.w2) > 0, ps.ay.w2)

ps.c <- prune_samples(sample_data(ps.f)$Horizon %in% c("C", "AC"),ps.f)
ps.c  <- prune_taxa(taxa_sums(ps.c) > 0, ps.c)


ps.c.w2 <- prune_samples(sample_data(ps.c)$Site != c("K2"), ps.c)
ps.c.w2 <- prune_taxa(taxa_sums(ps.c.w2) > 0, ps.c.w2)
```

```
des_w_simper <- function(ps, factor){
  require(DESeq2)
  require(vegan)
  require(tibble)
  require(phyloseq)
  
  diagdds = phyloseq_to_deseq2(ps, as.formula(paste( "~", factor)))                  
  diagdds = estimateSizeFactors(diagdds, type="poscounts")
  diagdds = estimateDispersions(diagdds, fitType = "local") 
  diagdds = DESeq(diagdds)
  samp <-sample_data(ps)
  aggdata <- t(aggregate.data.frame(as.data.frame(as.data.frame(t(diagdds@assays@data$mu))), by=list(samp[[factor]]), median))
  colnames(aggdata) <- aggdata[1,]
  aggdata <- aggdata[-1,]
  res = results(diagdds)
  res.df <- as.data.frame(res)
  nice <- cbind(res.df, as.data.frame(ps@tax_table@.Data[rownames(res.df),]), as.data.frame(aggdata)[rownames(res.df),])
}
```

```
des.ay <- des_w_simper(ps.ay.w2, "Site")
des.c <- des_w_simper(ps.c.w2, "Site")
```

#### K2AY <= K3AY

padj <= 0.1 sorted by baseMean

```
ps.23.ay <- prune_samples(sample_data(ps.f)$Description %in% c("K2-AY", "K3-AY"), ps.f)
ps.23.ay  <- prune_taxa(taxa_sums(ps.23.ay) > 0, ps.23.ay)


des.23.ay<- des_w_simper(ps.23.ay, "Site")
des.23.ay.little <- des.23.ay[des.23.ay$pvalue <= 0.05,]
des.23.ay.little <- des.23.ay.little[,c("class", "family", "genus", "species" , "log2FoldChange", "padj","baseMean", "K2", "K3")]
head(des.23.ay.little[order(des.23.ay.little$baseMean, decreasing = TRUE),], n=30)
```

```
##                              class                 family                        genus        species log2FoldChange           padj  baseMean          K2          K3
## Seq4                 Rubrobacteria      Rubrobacteriaceae                  Rubrobacter           <NA>     -0.6902170 0.209271408233 552.10332    723.0665    426.0813
## Seq13             Oxyphotobacteria                   <NA>                         <NA>           <NA>      4.8559580 0.000019353255 493.87263    30.86026   849.72593
## Seq5              Verrucomicrobiae Xiphinematobacteraceae Candidatus_Xiphinematobacter           <NA>      1.3170407 0.059831883913 216.39731    122.2671    289.6468
## Seq18                Rubrobacteria      Rubrobacteriaceae                  Rubrobacter           <NA>     -1.0520769 0.042319401759 180.25027    261.4547    119.8892
## Seq47                  Bacteroidia       Chitinophagaceae                         <NA>           <NA>     -0.7603520 0.210856388475 150.47342    201.2393    112.9573
## Seq32              Nitrososphaeria     Nitrososphaeraceae    Candidatus_Nitrososphaera           <NA>     -0.9347274 0.126653050798 120.36823   169.32997    84.22541
## Seq71                  Bacteroidia       Chitinophagaceae                         <NA>           <NA>     -0.8472082 0.133483564816 111.11914   152.44389    80.56849
## Seq101                     Bacilli         Planococcaceae                         <NA>           <NA>      6.2827587 0.000005837684 111.05479    2.625553  194.361383
## Seq51               Entotheonellia      Entotheonellaceae     Candidatus_Entotheonella           <NA>     -1.3706198 0.028424766409  92.50186   144.99861    53.31587
## Seq136                     Bacilli         Planococcaceae               Lysinibacillus           <NA>      6.1424624 0.000002217777  91.77094    2.388674  160.440006
## Seq55  Blastocatellia_(Subgroup_4)      Blastocatellaceae                         <NA>           <NA>     -0.9315486 0.204897921856  85.02177   119.61637    59.62891
## Seq106         Gammaproteobacteria       Burkholderiaceae                  Ramlibacter           <NA>     -1.1464142 0.035831022687  82.29999   122.28875    52.52569
## Seq178                 Bacteroidia       Chitinophagaceae                         <NA>           <NA>     -1.5082661 0.034873009920  74.52068   120.36027    40.22908
## Seq145                 Bacteroidia       Chitinophagaceae              Flavisolibacter ginsengiterrae     -1.0268045 0.124036315475  70.68608    101.9850     47.5913
## Seq172                 Bacteroidia       Chitinophagaceae                         <NA>           <NA>     -1.5664116 0.048936849544  65.18515   106.62111    34.22919
## Seq322                 Bacteroidia       Chitinophagaceae                         <NA>           <NA>      9.2041431 0.000928666722  61.01939   0.1923917 107.8945958
## Seq137            Verrucomicrobiae    Chthoniobacteraceae               Chthoniobacter           <NA>     -0.9083007 0.212757140169  57.27677    79.89432    40.47440
## Seq391         Alphaproteobacteria           Mitochondria                         <NA>           <NA>      5.4420457 0.055577708860  55.28865    2.322594   96.002522
## Seq29             Verrucomicrobiae    Chthoniobacteraceae       Candidatus_Udaeobacter       copiosus      1.7755367 0.002514736073  52.35507    23.10500    75.21182
## Seq223         Alphaproteobacteria        Azospirillaceae                  Skermanella           <NA>     -1.1382169 0.037169053170  49.66916    73.70392    31.83783
## Seq176                 Bacteroidia       Chitinophagaceae                   Flavitalea           <NA>     -1.3939973 0.032824040321  47.52926    74.81275    27.06643
## Seq339                  Clostridia  Peptostreptococcaceae                   Romboutsia           <NA>      3.2444035 0.011860686411  45.93065    8.313558   74.910036
## Seq299 Blastocatellia_(Subgroup_4)      Blastocatellaceae            Stenotrophobacter           <NA>     -1.1114093 0.188965694319  44.42489    65.34386    28.75594
## Seq264             Thermoleophilia   Solirubrobacteraceae              Solirubrobacter           <NA>     -1.3861085 0.071655586821  43.13225    67.80083    24.66409
## Seq252               Rubrobacteria      Rubrobacteriaceae                  Rubrobacter           <NA>     -1.1246927 0.084442223123  42.40841    62.79985    27.38310
## Seq379              Actinobacteria     Pseudonocardiaceae               Pseudonocardia           <NA>      1.9727596 0.037169053170  42.27788    16.65109    62.14309
## Seq122              Actinobacteria     Micromonosporaceae                 Actinoplanes           <NA>      1.7981053 0.017903446463  42.08335    18.33308    60.61909
## Seq8              Verrucomicrobiae    Chthoniobacteraceae       Candidatus_Udaeobacter           <NA>      2.1886471 0.004059966740  41.63250    14.46773    62.71034
## Seq406         Alphaproteobacteria       Beijerinckiaceae                   Microvirga           <NA>     -2.0506494 0.006337265674  40.86693    73.07056    16.76972
## Seq375              Actinobacteria      Microbacteriaceae               Microbacterium           <NA>      1.5002450 0.177850598912  40.12390    20.58712    55.37385
```

#### K3C <= K2C

padj <= 0.05 sorted by baseMean

```
ps.32.c <- prune_samples(sample_data(ps.f)$Description %in% c("K3-C", "K2-C"), ps.f)
ps.32.c  <- prune_taxa(taxa_sums(ps.32.c) > 0, ps.32.c)


des.32.c<- des_w_simper(ps.32.c, "Site")
des.32.c.little <- des.32.c[des.32.c$pvalue <= 0.05,]
des.32.c.little <- des.32.c.little[,c("class", "family", "genus", "species" , "log2FoldChange", "padj","baseMean")]
head(des.32.c.little[order(des.32.c.little$baseMean, decreasing = TRUE),], n=30)
```

```
##                              class                 family                        genus  species log2FoldChange             padj  baseMean
## Seq8              Verrucomicrobiae    Chthoniobacteraceae       Candidatus_Udaeobacter     <NA>      2.2297154 0.00000001256169 420.66505
## Seq5              Verrucomicrobiae Xiphinematobacteraceae Candidatus_Xiphinematobacter     <NA>      1.8583671 0.00186389510441 371.34034
## Seq19  Blastocatellia_(Subgroup_4)       Pyrinomonadaceae                         RB41     <NA>      0.9456689 0.23072471255003 286.64823
## Seq16              Nitrososphaeria     Nitrososphaeraceae                         <NA>     <NA>     -1.0099998 0.08206581914651 209.66124
## Seq29             Verrucomicrobiae    Chthoniobacteraceae       Candidatus_Udaeobacter copiosus      1.3397909 0.00590735303239 200.00985
## Seq7                   Bacteroidia        Microscillaceae                         <NA>     <NA>     -1.2879384 0.01048760974141 162.85297
## Seq23          Alphaproteobacteria      Xanthobacteraceae                         <NA>     <NA>      0.7831766 0.17323010048667 140.84314
## Seq31  Blastocatellia_(Subgroup_4)      Blastocatellaceae                         <NA>     <NA>      1.4467723 0.00093896981311 126.76307
## Seq6               Thermoleophilia                  67-14                         <NA>     <NA>      0.7572074 0.24581532592615 120.60852
## Seq80  Blastocatellia_(Subgroup_4)       Pyrinomonadaceae                         RB41     <NA>      1.4225795 0.05743886785235 117.50030
## Seq28              Thermoleophilia                  67-14                         <NA>     <NA>      1.4682131 0.00182092701751  96.62984
## Seq44             Verrucomicrobiae Xiphinematobacteraceae Candidatus_Xiphinematobacter     <NA>      1.4848378 0.00645008803754  95.42536
## Seq32              Nitrososphaeria     Nitrososphaeraceae    Candidatus_Nitrososphaera     <NA>     -0.7303119 0.23523201173415  79.76755
## Seq53                  Bacteroidia       Chitinophagaceae                         <NA>     <NA>     -2.2464986 0.00279723903693  78.00566
## Seq242                     Bacilli         Planococcaceae                         <NA>     <NA>      2.1746283 0.00186389510441  58.89644
## Seq204                 Bacteroidia       Chitinophagaceae                         <NA>     <NA>     -1.2988222 0.17042495835560  55.06039
## Seq330             Nitrososphaeria     Nitrososphaeraceae                         <NA>     <NA>     -1.1385370 0.18705978934018  51.26132
## Seq112                  Subgroup_6                   <NA>                         <NA>     <NA>      1.0706255 0.06173710439958  49.59135
## Seq320              Acidimicrobiia                   <NA>                         <NA>     <NA>      1.7732765 0.00068955300171  48.71683
## Seq193         Gammaproteobacteria                SC-I-84                         <NA>     <NA>      2.2399658 0.00066186895223  48.34003
## Seq498              Actinobacteria     Pseudonocardiaceae              Actinophytocola     <NA>      1.1583362 0.24241147243214  45.26491
## Seq316                 Subgroup_18                   <NA>                         <NA>     <NA>      1.3198681 0.09803503262804  45.01591
## Seq246               Phycisphaerae      WD2101_soil_group                         <NA>     <NA>      1.4064123 0.09803503262804  44.61771
## Seq218              Acidimicrobiia                   <NA>                         <NA>     <NA>      1.5130077 0.04357995121887  44.59771
## Seq90               Actinobacteria   Propionibacteriaceae                 Microlunatus     <NA>     -1.8148877 0.00590735303239  39.11818
## Seq445                 Bacteroidia      Hymenobacteraceae                Adhaeribacter     <NA>      4.6952928 0.00000003669410  39.05398
## Seq283            Verrucomicrobiae Xiphinematobacteraceae Candidatus_Xiphinematobacter     <NA>      2.3056832 0.00068955300171  37.57106
## Seq292                        TK10                   <NA>                         <NA>     <NA>      1.2532720 0.07446400005776  37.48681
## Seq440            Verrucomicrobiae    Chthoniobacteraceae               Chthoniobacter     <NA>      1.3838335 0.07740415140092  35.75134
## Seq636                        TK10                   <NA>                         <NA>     <NA>      1.0773684 0.21950976902545  34.67245
```

#### K6AY <= K3AY

padj <= 0.05 sorted by baseMean

```
ps.63.ay <- prune_samples(sample_data(ps.f)$Description %in% c("K6-AY", "K3-AY"), ps.f)
ps.63.ay  <- prune_taxa(taxa_sums(ps.63.ay) > 0, ps.63.ay)


des.63.ay<- des_w_simper(ps.63.ay, "Site")
des.63.ay.little <- des.63.ay[des.63.ay$pvalue <= 0.05,]
des.63.ay.little <- des.63.ay.little[,c("class", "family", "genus", "species" , "log2FoldChange", "padj","baseMean")]
head(des.63.ay.little[order(des.63.ay.little$baseMean, decreasing = TRUE),],n=30)
```

```
##                              class                       family                        genus    species log2FoldChange         padj  baseMean
## Seq1               Nitrososphaeria           Nitrososphaeraceae                         <NA>       <NA>     -1.2496476 3.182685e-03 488.51004
## Seq13             Oxyphotobacteria                         <NA>                         <NA>       <NA>     -9.9181301 4.510890e-11 424.40545
## Seq11  Blastocatellia_(Subgroup_4)             Pyrinomonadaceae                         RB41       <NA>      5.0511850 7.488906e-42 313.87699
## Seq20              Nitrososphaeria           Nitrososphaeraceae     Candidatus_Nitrocosmicus       <NA>      6.9156269 2.754011e-56 282.03682
## Seq4                 Rubrobacteria            Rubrobacteriaceae                  Rubrobacter       <NA>     -2.6254880 3.249839e-13 253.55292
## Seq5              Verrucomicrobiae       Xiphinematobacteraceae Candidatus_Xiphinematobacter       <NA>     -3.2376422 3.432189e-17 164.70349
## Seq12              Nitrososphaeria           Nitrososphaeraceae                         <NA>       <NA>     -0.9019232 5.342809e-02 159.41671
## Seq16              Nitrososphaeria           Nitrososphaeraceae                         <NA>       <NA>      0.8744518 3.450204e-02 152.85693
## Seq10          Gammaproteobacteria             Burkholderiaceae                         <NA>       <NA>     -1.2526268 6.819683e-05 150.76521
## Seq37                  Bacteroidia             Chitinophagaceae                         <NA>       <NA>     -3.9428088 9.913543e-10 129.36923
## Seq35               Acidobacteriia Solibacteraceae_(Subgroup_3)                   Bryobacter       <NA>     -1.0277809 2.845628e-02 123.92470
## Seq15          Deltaproteobacteria                         <NA>                         <NA>       <NA>     -1.5096323 4.587891e-03 115.02011
## Seq101                     Bacilli               Planococcaceae                         <NA>       <NA>     -3.4051953 5.496192e-04 106.18617
## Seq2               Nitrososphaeria           Nitrososphaeraceae                         <NA>       <NA>      1.1445372 8.036686e-03 105.50699
## Seq14          Alphaproteobacteria            Xanthobacteraceae               Bradyrhizobium       <NA>      1.3468044 1.576730e-03  97.39025
## Seq18                Rubrobacteria            Rubrobacteriaceae                  Rubrobacter       <NA>     -0.8043396 1.145121e-02  96.29822
## Seq17                      Bacilli                         <NA>                         <NA>       <NA>     -1.2237518 3.179254e-02  95.98438
## Seq53                  Bacteroidia             Chitinophagaceae                         <NA>       <NA>      1.9361306 5.107019e-05  94.40378
## Seq136                     Bacilli               Planococcaceae               Lysinibacillus       <NA>     -3.6678868 4.328289e-05  86.62313
## Seq165            Oxyphotobacteria                         <NA>                         <NA>       <NA>      2.7029029 3.429441e-03  84.89485
## Seq119 Blastocatellia_(Subgroup_4)             Pyrinomonadaceae                         RB41       <NA>      3.1701006 3.065906e-12  81.77933
## Seq60  Blastocatellia_(Subgroup_4)            Blastocatellaceae                  Aridibacter famidurans     -5.1366962 3.750045e-22  78.87550
## Seq56          Deltaproteobacteria                         <NA>                         <NA>       <NA>     -0.9003433 3.409197e-02  77.36357
## Seq34              Thermoleophilia                         <NA>                         <NA>       <NA>     -4.3318491 1.413236e-15  76.11529
## Seq41  Blastocatellia_(Subgroup_4)             Pyrinomonadaceae                         RB41       <NA>      1.2446784 6.541400e-04  75.32734
## Seq31  Blastocatellia_(Subgroup_4)            Blastocatellaceae                         <NA>       <NA>     -2.3942744 5.258145e-13  72.39609
## Seq30          Gammaproteobacteria          Steroidobacteraceae               Steroidobacter     uvarum      1.9954990 3.503873e-08  71.89920
## Seq94                   Subgroup_6                         <NA>                         <NA>       <NA>      1.9149295 1.092631e-07  70.41849
## Seq161                  Nitrospira               Nitrospiraceae                   Nitrospira   japonica      3.7894569 1.316945e-16  68.69838
## Seq76                Rubrobacteria            Rubrobacteriaceae                  Rubrobacter       <NA>     -2.2544902 3.666089e-07  67.33751
```

#### K6AY <= K1AY

padj <= 0.05 sorted by baseMean

```
ps.61.ay <- prune_samples(sample_data(ps.f)$Description %in% c("K6-AY", "K1-AY"), ps.f)
ps.61.ay  <- prune_taxa(taxa_sums(ps.61.ay) > 0, ps.61.ay)


des.61.ay<- des_w_simper(ps.61.ay, "Site")
des.61.ay.little <- des.61.ay[des.61.ay$pvalue <= 0.05,]
des.61.ay.little <- des.61.ay.little[,c("class", "family", "genus", "species" , "log2FoldChange", "padj","baseMean")]
head(des.61.ay.little[order(des.61.ay.little$baseMean, decreasing = TRUE),], n=30)
```

```
##                              class               family                     genus        species log2FoldChange         padj  baseMean
## Seq2               Nitrososphaeria   Nitrososphaeraceae                      <NA>           <NA>     -1.9600443 4.935722e-06 343.93501
## Seq3                       Bacilli                 <NA>                      <NA> longiquaesitum      9.4107381 3.671233e-34 337.93909
## Seq11  Blastocatellia_(Subgroup_4)     Pyrinomonadaceae                      RB41           <NA>      4.5317267 1.414452e-48 309.69853
## Seq20              Nitrososphaeria   Nitrososphaeraceae  Candidatus_Nitrocosmicus           <NA>      4.1698488 1.196950e-43 287.02707
## Seq6               Thermoleophilia                67-14                      <NA>           <NA>     -5.2249513 1.018728e-45 187.53285
## Seq30          Gammaproteobacteria  Steroidobacteraceae            Steroidobacter         uvarum     -0.8942872 1.013325e-03 160.02997
## Seq7                   Bacteroidia      Microscillaceae                      <NA>           <NA>      2.3783229 3.001192e-18 150.13146
## Seq1               Nitrososphaeria   Nitrososphaeraceae                      <NA>           <NA>      7.6266714 1.994774e-35 140.86738
## Seq21              Nitrososphaeria   Nitrososphaeraceae                      <NA>           <NA>     -1.5967012 1.137861e-06 123.41872
## Seq36              Thermoleophilia Solirubrobacteraceae           Solirubrobacter           <NA>     -4.4758979 4.661652e-33 104.72293
## Seq16              Nitrososphaeria   Nitrososphaeraceae                      <NA>           <NA>      9.9985217 1.549756e-23  95.99914
## Seq24                         <NA>                 <NA>                      <NA>           <NA>     -2.2959230 9.704926e-09  93.65713
## Seq42          Alphaproteobacteria       Reyranellaceae                Reyranella           <NA>     -2.7515738 6.308853e-22  88.06108
## Seq50               Actinobacteria   Micromonosporaceae                    Asanoa           <NA>     -0.6003106 4.285014e-02  83.69577
## Seq46          Alphaproteobacteria    Xanthobacteraceae                      <NA>           <NA>     -2.1148176 7.024057e-13  79.63385
## Seq40               Acidimicrobiia   Ilumatobacteraceae                      <NA>           <NA>     -2.5855485 7.243064e-20  79.40673
## Seq33              Nitrososphaeria   Nitrososphaeraceae                      <NA>           <NA>     -7.6638276 7.049111e-21  78.91617
## Seq48              Thermoleophilia Solirubrobacteraceae           Solirubrobacter           <NA>     -1.0144864 3.249757e-04  78.00737
## Seq25               Actinobacteria Propionibacteriaceae              Microlunatus           <NA>     -8.0594616 2.264272e-18  77.47479
## Seq81               Acidimicrobiia                 <NA>                      <NA>           <NA>     -0.8494601 4.815188e-02  74.60126
## Seq49          Gammaproteobacteria    Nitrosomonadaceae                      MND1           <NA>     -9.6831806 2.148062e-20  74.20715
## Seq53                  Bacteroidia     Chitinophagaceae                      <NA>           <NA>      9.0250517 3.707050e-19  72.91090
## Seq165            Oxyphotobacteria                 <NA>                      <NA>           <NA>      9.5871242 1.074578e-11  72.14437
## Seq119 Blastocatellia_(Subgroup_4)     Pyrinomonadaceae                      RB41           <NA>      6.6721824 2.987569e-27  72.06971
## Seq57             Verrucomicrobiae  Chthoniobacteraceae    Candidatus_Udaeobacter           <NA>     -2.5828842 4.443107e-20  71.75281
## Seq94                   Subgroup_6                 <NA>                      <NA>           <NA>      2.0003699 8.282151e-12  67.80215
## Seq169             Nitrososphaeria   Nitrososphaeraceae                      <NA>           <NA>      1.3963539 2.047760e-06  66.32096
## Seq161                  Nitrospira       Nitrospiraceae                Nitrospira       japonica      4.0569890 3.303835e-28  65.94252
## Seq93          Gammaproteobacteria                 <NA>                      <NA>           <NA>     -1.5841491 1.639893e-05  65.67189
## Seq32              Nitrososphaeria   Nitrososphaeraceae Candidatus_Nitrososphaera           <NA>      7.6847956 1.237219e-16  62.73745
```
