# Supplementary figures and images for: Microbiomes of different ages in Rendzic Leptosols in the Crimean Peninsula
Source: PeerJ. 2021 Feb 18;9:e10871. doi: 10.7717/peerj.10871 (PMC7897411; doi:10.7717/peerj.10871)

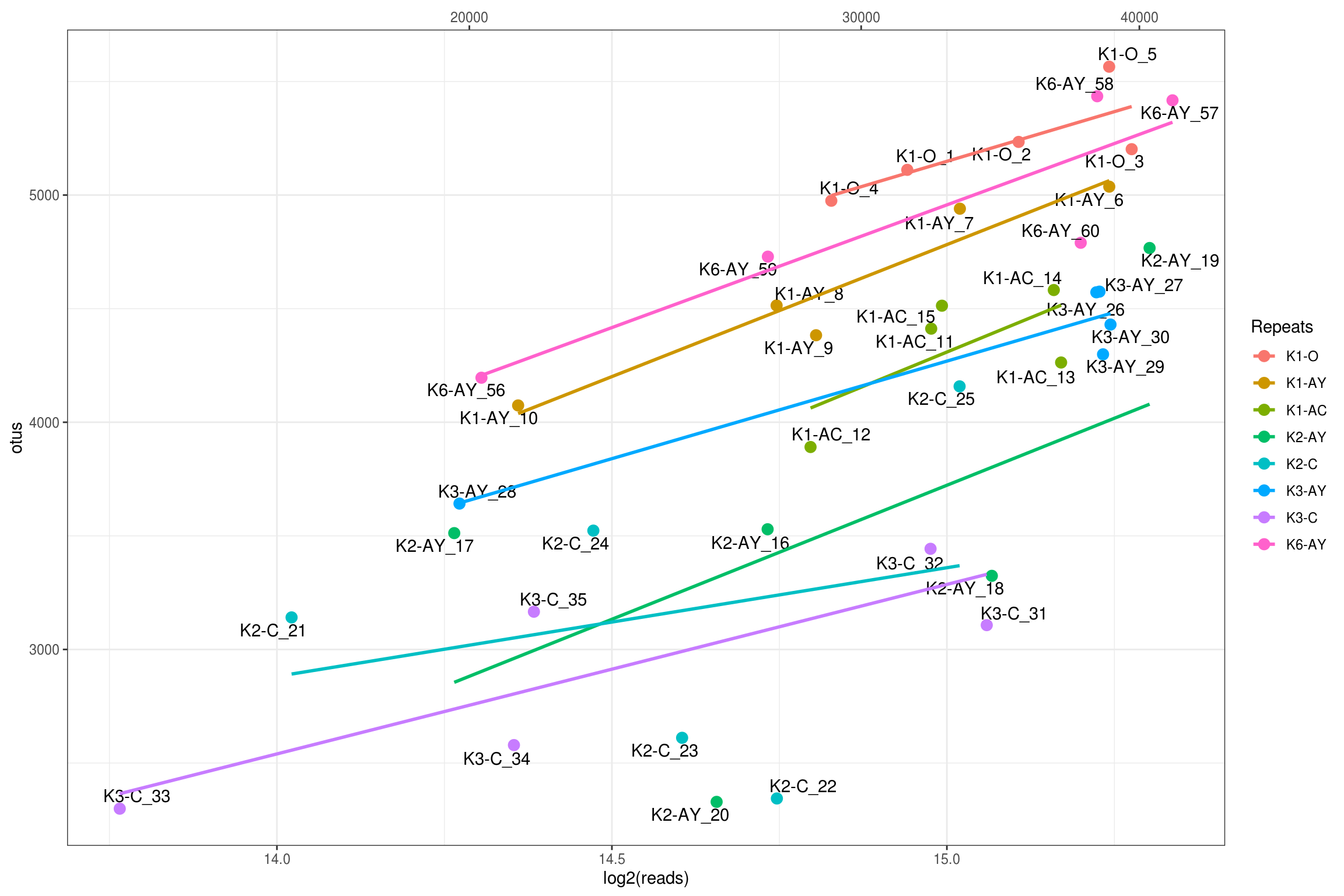

Supplement: Supplemental Information 3 — Shows ratio of observed OTU (phylotypes) and depth of sequencing. Each colour represents all replicates from a certain soil horizon in a certain site. [file peerj-09-10871-s003.png]

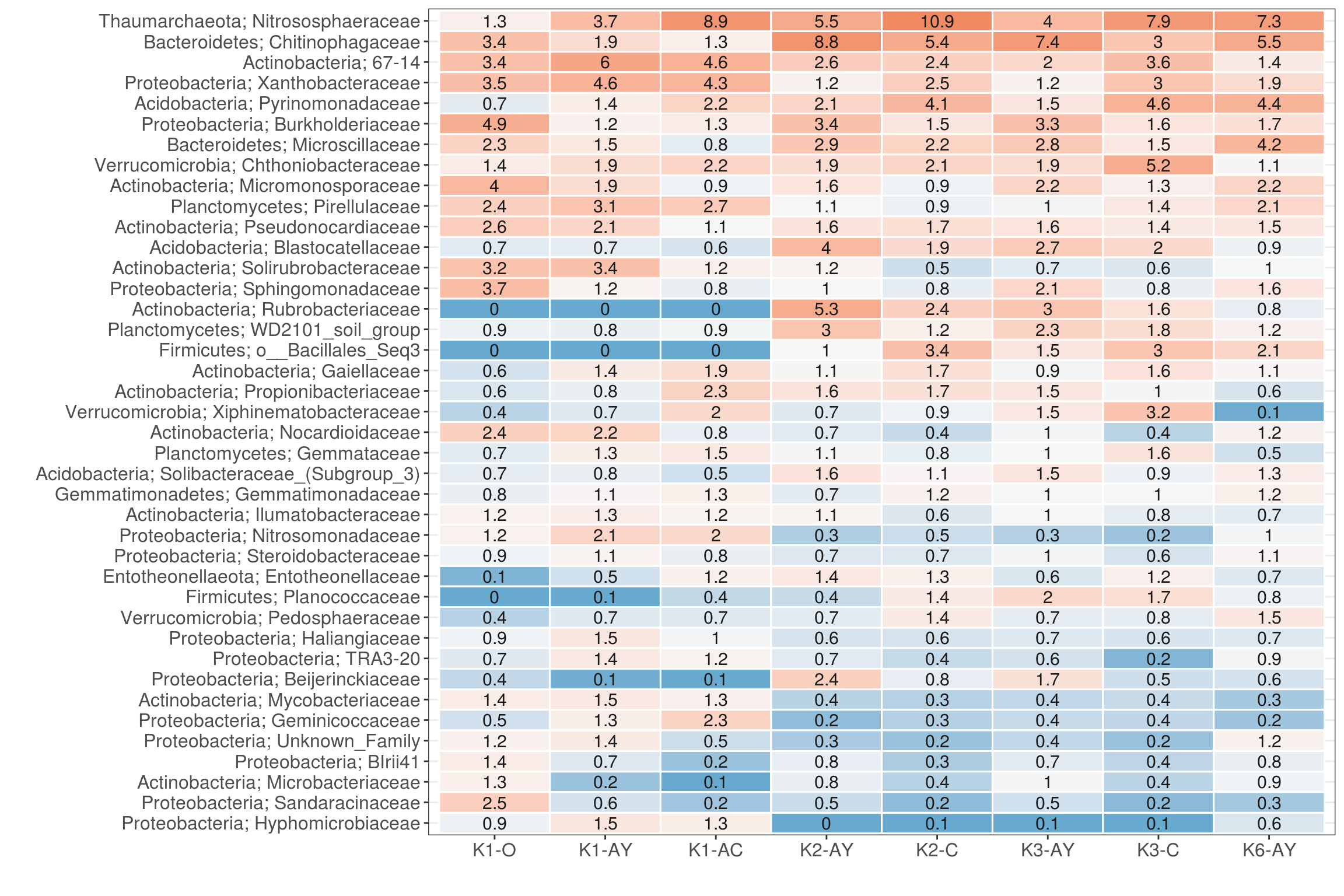

Supplement: Supplemental Information 4 — Orange is more abundant, blue –less. [file peerj-09-10871-s004.png]
