# Supplementary material for: Microbiomes of different ages in Rendzic Leptosols in the Crimean Peninsula
Source: PeerJ. 2021 Feb 18;9:e10871. doi: 10.7717/peerj.10871 (PMC7897411; doi:10.7717/peerj.10871)
Supplement: Supplemental Information 5 [file peerj-09-10871-s005.docx]

| Factor | R2 | Pr(>F) |
| --- | --- | --- |
| Residual | 0.5697825 | NA |
| Horizon:Site | 0.3321851 | 0.001 |
| Horizon:N_tot_ | 0.1637269 | 0.001 |
| Horizon:K_2_O | 0.1478728 | 0.001 |
| Horizon:pH | 0.1377684 | 0.001 |
| Horizon:P_2_O_5_ | 0.1126650 | 0.001 |
| Horizon:C_carb_ | 0.1097756 | 0.001 |
| Horizon:TOC | 0.1051342 | 0.001 |
