# Supplementary material for: Microbiomes of different ages in Rendzic Leptosols in the Crimean Peninsula
Source: PeerJ. 2021 Feb 18;9:e10871. doi: 10.7717/peerj.10871 (PMC7897411; doi:10.7717/peerj.10871)
Supplement: Supplemental Information 6 [file peerj-09-10871-s006.docx]

|  | class | family | genus/species | log2FoldChange | padj | baseMean |
| --- | --- | --- | --- | --- | --- | --- |
| K3-AY > K2-AY | | | | | | |
| Seq4 | Rubrobacteria | Rubrobacteriaceae | Rubrobacter | -0.690217 | 2.09E-01 | 552.10332 |
| Seq13 | Oxyphotobacteria | <NA> | <NA> | **4.855958** | 1.94E-05 | 493.87263 |
| Seq5 | Verrucomicrobiae | Xiphinematobacteraceae | Candidatus_Xiphinematobacter | 1.3170407 | 5.98E-02 | 216.39731 |
| Seq18 | Rubrobacteria | Rubrobacteriaceae | Rubrobacter | **-1.0520769** | 4.23E-02 | 180.25027 |
| Seq47 | Bacteroidia | Chitinophagaceae | <NA> | -0.760352 | 2.11E-01 | 150.47342 |
| Seq32 | Nitrososphaeria | Nitrososphaeraceae | Candidatus_Nitrososphaera | -0.9347274 | 1.27E-01 | 120.36823 |
| Seq71 | Bacteroidia | Chitinophagaceae | <NA> | -0.8472082 | 1.33E-01 | 111.11914 |
| Seq101 | Bacilli | Planococcaceae | <NA> | **6.2827587** | 5.84E-06 | 111.05479 |
| Seq51 | Entotheonellia | Entotheonellaceae | Candidatus_Entotheonella | **-1.3706198** | 2.84E-02 | 92.50186 |
| Seq136 | Bacilli | Planococcaceae | Lysinibacillus | **6.1424624** | 2.22E-06 | 91.77094 |
| Seq55 | Blastocatellia_(Subgroup_4) | Blastocatellaceae | <NA> | -0.9315486 | 2.05E-01 | 85.02177 |
| Seq106 | Gammaproteobacteria | Burkholderiaceae | Ramlibacter | **-1.1464142** | 3.58E-02 | 82.29999 |
| Seq178 | Bacteroidia | Chitinophagaceae | <NA> | **-1.5082661** | 3.49E-02 | 74.52068 |
| Seq145 | Bacteroidia | Chitinophagaceae | Flavisolibacter ginsengiterrae | -1.0268045 | 1.24E-01 | 70.68608 |
| Seq172 | Bacteroidia | Chitinophagaceae | <NA> | **-1.5664116** | 4.89E-02 | 65.18515 |
| Seq322 | Bacteroidia | Chitinophagaceae | <NA> | **9.2041431** | 9.29E-04 | 61.01939 |
| Seq137 | Verrucomicrobiae | Chthoniobacteraceae | Chthoniobacter | -0.9083007 | 2.13E-01 | 57.27677 |
| Seq391 | Alphaproteobacteria | Mitochondria | <NA> | 5.4420457 | 5.56E-02 | 55.28865 |
| Seq29 | Verrucomicrobiae | Chthoniobacteraceae | Candidatus_Udaeobacter copiosus | **1.7755367** | 2.51E-03 | 52.35507 |
| Seq223 | Alphaproteobacteria | Azospirillaceae | Skermanella | **-1.1382169** | 3.72E-02 | 49.66916 |
| Seq176 | Bacteroidia | Chitinophagaceae | Flavitalea | **-1.3939973** | 3.28E-02 | 47.52926 |
| Seq339 | Clostridia | Peptostreptococcaceae | Romboutsia | **3.2444035** | 1.19E-02 | 45.93065 |
| Seq299 | Blastocatellia_(Subgroup_4) | Blastocatellaceae | Stenotrophobacter | -1.1114093 | 1.89E-01 | 44.42489 |
| Seq264 | Thermoleophilia | Solirubrobacteraceae | Solirubrobacter | -1.3861085 | 7.17E-02 | 43.13225 |
| Seq252 | Rubrobacteria | Rubrobacteriaceae | Rubrobacter | -1.1246927 | 8.44E-02 | 42.40841 |
| Seq379 | Actinobacteria | Pseudonocardiaceae | Pseudonocardia | **1.9727596** | 3.72E-02 | 42.27788 |
| Seq122 | Actinobacteria | Micromonosporaceae | Actinoplanes | **1.7981053** | 1.79E-02 | 42.08335 |
| Seq8 | Verrucomicrobiae | Chthoniobacteraceae | Candidatus_Udaeobacter | **2.1886471** | 4.06E-03 | 41.63250 |
| Seq406 | Alphaproteobacteria | Beijerinckiaceae | Microvirga | **-2.0506494** | 6.34E-03 | 40.86693 |
| Seq375 | Actinobacteria | Microbacteriaceae | Microbacterium | 1.500245 | 1.78E-01 | 40.12390 |
|  |  |  |  |  |  |  |
| K3-C > K2-C | | | | | | |
| Seq8 | Verrucomicrobiae | Chthoniobacteraceae | Candidatus_Udaeobacter | **2.2297154** | 1.26E-08 | 420.66505 |
| Seq5 | Verrucomicrobiae | Xiphinematobacteraceae | Candidatus_Xiphinematobacter | **1.8583671** | 1.86E-03 | 371.34034 |
| Seq19 | Blastocatellia_(Subgroup_4) | Pyrinomonadaceae | RB41 | 0.9456689 | 2.31E-01 | 286.64823 |
| Seq16 | Nitrososphaeria | Nitrososphaeraceae | <NA> | -1.0099998 | 8.21E-02 | 209.66124 |
| Seq29 | Verrucomicrobiae | Chthoniobacteraceae | Candidatus_Udaeobacter copiosus | **1.3397909** | 5.91E-03 | 200.00985 |
| Seq7 | Bacteroidia | Microscillaceae | <NA> | **-1.2879384** | 1.05E-02 | 162.85297 |
| Seq23 | Alphaproteobacteria | Xanthobacteraceae | <NA> | 0.7831766 | 1.73E-01 | 140.84314 |
| Seq31 | Blastocatellia_(Subgroup_4) | Blastocatellaceae | <NA> | **1.4467723** | 9.39E-04 | 126.76307 |
| Seq6 | Thermoleophilia | 67-14 | <NA> | 0.7572074 | 2.46E-01 | 120.60852 |
| Seq80 | Blastocatellia_(Subgroup_4) | Pyrinomonadaceae | RB41 | 1.4225795 | 5.74E-02 | 117.50030 |
| Seq28 | Thermoleophilia | 67-14 | <NA> | **1.4682131** | 1.82E-03 | 96.62984 |
| Seq44 | Verrucomicrobiae | Xiphinematobacteraceae | Candidatus_Xiphinematobacter | **1.4848378** | 6.45E-03 | 95.42536 |
| Seq32 | Nitrososphaeria | Nitrososphaeraceae | Candidatus_Nitrososphaera | -0.7303119 | 2.35E-01 | 79.76755 |
| Seq53 | Bacteroidia | Chitinophagaceae | <NA> | **-2.2464986** | 2.80E-03 | 78.00566 |
| Seq242 | Bacilli | Planococcaceae | <NA> | **2.1746283** | 1.86E-03 | 58.89644 |
| Seq204 | Bacteroidia | Chitinophagaceae | <NA> | -1.2988222 | 1.70E-01 | 55.06039 |
| Seq330 | Nitrososphaeria | Nitrososphaeraceae | <NA> | -1.138537 | 1.87E-01 | 51.26132 |
| Seq112 | Subgroup_6 | <NA> | <NA> | 1.0706255 | 6.17E-02 | 49.59135 |
| Seq320 | Acidimicrobiia | <NA> | <NA> | **1.7732765** | 6.90E-04 | 48.71683 |
| Seq193 | Gammaproteobacteria | SC-I-84 | <NA> | **2.2399658** | 6.62E-04 | 48.34003 |
| Seq498 | Actinobacteria | Pseudonocardiaceae | Actinophytocola | 1.1583362 | 2.42E-01 | 45.26491 |
| Seq316 | Subgroup_18 | <NA> | <NA> | 1.3198681 | 9.80E-02 | 45.01591 |
| Seq246 | Phycisphaerae | WD2101_soil_group | <NA> | 1.4064123 | 9.80E-02 | 44.61771 |
| Seq218 | Acidimicrobiia | <NA> | <NA> | **1.5130077** | 4.36E-02 | 44.59771 |
| Seq90 | Actinobacteria | Propionibacteriaceae | Microlunatus | **-1.8148877** | 5.91E-03 | 39.11818 |
| Seq445 | Bacteroidia | Hymenobacteraceae | Adhaeribacter | **4.6952928** | 3.67E-08 | 39.05398 |
| Seq283 | Verrucomicrobiae | Xiphinematobacteraceae | Candidatus_Xiphinematobacter | **2.3056832** | 6.90E-04 | 37.57106 |
| Seq292 | TK10 | <NA> | <NA> | 1.253272 | 7.45E-02 | 37.48681 |
| Seq440 | Verrucomicrobiae | Chthoniobacteraceae | Chthoniobacter | 1.3838335 | 7.74E-02 | 35.75134 |
| Seq636 | TK10 | <NA> | <NA> | 1.0773684 | 2.20E-01 | 34.67245 |
|  |  |  |  |  |  |  |
| K6-AY > K3-AY | | | | | | |
| Seq1 | Nitrososphaeria | Nitrososphaeraceae | <NA> | **-1.2496476** | 3.18E-03 | 488.51004 |
| Seq13 | Oxyphotobacteria | <NA> | <NA> | **-9.9181301** | 4.51E-11 | 424.40545 |
| Seq11 | Blastocatellia_(Subgroup_4) | Pyrinomonadaceae | RB41 | **5.051185** | 7.49E-42 | 313.87699 |
| Seq20 | Nitrososphaeria | Nitrososphaeraceae | Candidatus_Nitrocosmicus | **6.9156269** | 2.75E-56 | 282.03682 |
| Seq4 | Rubrobacteria | Rubrobacteriaceae | Rubrobacter | **-2.625488** | 3.25E-13 | 253.55292 |
| Seq5 | Verrucomicrobiae | Xiphinematobacteraceae | Candidatus_Xiphinematobacter | **-3.2376422** | 3.43E-17 | 164.70349 |
| Seq12 | Nitrososphaeria | Nitrososphaeraceae | <NA> | -0.9019232 | 5.34E-02 | 159.41671 |
| Seq16 | Nitrososphaeria | Nitrososphaeraceae | <NA> | **0.8744518** | 3.45E-02 | 152.85693 |
| Seq10 | Gammaproteobacteria | Burkholderiaceae | <NA> | **-1.2526268** | 6.82E-05 | 150.76521 |
| Seq37 | Bacteroidia | Chitinophagaceae | <NA> | **-3.9428088** | 9.91E-10 | 129.36923 |
| Seq35 | Acidobacteriia | Solibacteraceae_(Subgroup_3) | Bryobacter | **-1.0277809** | 2.85E-02 | 123.92470 |
| Seq15 | Deltaproteobacteria | <NA> | <NA> | **-1.5096323** | 4.59E-03 | 115.02011 |
| Seq101 | Bacilli | Planococcaceae | <NA> | **-3.4051953** | 5.50E-04 | 106.18617 |
| Seq2 | Nitrososphaeria | Nitrososphaeraceae | <NA> | **1.1445372** | 8.04E-03 | 105.50699 |
| Seq14 | Alphaproteobacteria | Xanthobacteraceae | Bradyrhizobium | **1.3468044** | 1.58E-03 | 97.39025 |
| Seq18 | Rubrobacteria | Rubrobacteriaceae | Rubrobacter | **-0.8043396** | 1.15E-02 | 96.29822 |
| Seq17 | Bacilli | <NA> | <NA> | **-1.2237518** | 3.18E-02 | 95.98438 |
| Seq53 | Bacteroidia | Chitinophagaceae | <NA> | **1.9361306** | 5.11E-05 | 94.40378 |
| Seq136 | Bacilli | Planococcaceae | Lysinibacillus | **-3.6678868** | 4.33E-05 | 86.62313 |
| Seq165 | Oxyphotobacteria | <NA> | <NA> | **2.7029029** | 3.43E-03 | 84.89485 |
| Seq119 | Blastocatellia_(Subgroup_4) | Pyrinomonadaceae | RB41 | **3.1701006** | 3.07E-12 | 81.77933 |
| Seq60 | Blastocatellia_(Subgroup_4) | Blastocatellaceae | Aridibacter famidurans | **-5.1366962** | 3.75E-22 | 78.87550 |
| Seq56 | Deltaproteobacteria | <NA> | <NA> | **-0.9003433** | 3.41E-02 | 77.36357 |
| Seq34 | Thermoleophilia | <NA> | <NA> | **-4.3318491** | 1.41E-15 | 76.11529 |
| Seq41 | Blastocatellia_(Subgroup_4) | Pyrinomonadaceae | RB41 | **1.2446784** | 6.54E-04 | 75.32734 |
| Seq31 | Blastocatellia_(Subgroup_4) | Blastocatellaceae | <NA> | **-2.3942744** | 5.26E-13 | 72.39609 |
| Seq30 | Gammaproteobacteria | Steroidobacteraceae | Steroidobacter famidurans | **1.995499** | 3.50E-08 | 71.89920 |
| Seq94 | Subgroup_6 | <NA> | <NA> | **1.9149295** | 1.09E-07 | 70.41849 |
| Seq161 | Nitrospira | Nitrospiraceae | Nitrospira japonica | **3.7894569** | 1.32E-16 | 68.69838 |
| Seq76 | Rubrobacteria | Rubrobacteriaceae | Rubrobacter | **-2.2544902** | 3.67E-07 | 67.33751 |
|  |  |  |  |  |  |  |
| K6-AY > K1-AY | | | | | | |
| Seq2 | Nitrososphaeria | Nitrososphaeraceae | <NA> | **-1.9600443** | 4.94E-06 | 343.93501 |
| Seq3 | Bacilli | <NA> | Bacillus longiquaestium | **9.4107381** | 3.67E-34 | 337.93909 |
| Seq11 | Blastocatellia_(Subgroup_4) | Pyrinomonadaceae | RB41 | **4.5317267** | 1.41E-48 | 309.69853 |
| Seq20 | Nitrososphaeria | Nitrososphaeraceae | Candidatus_Nitrocosmicus | **4.1698488** | 1.20E-43 | 287.02707 |
| Seq6 | Thermoleophilia | 67-14 | <NA> | **-5.2249513** | 1.02E-45 | 187.53285 |
| Seq30 | Gammaproteobacteria | Steroidobacteraceae | Steroidobacter uvarum | **-0.8942872** | 1.01E-03 | 160.02997 |
| Seq7 | Bacteroidia | Microscillaceae | <NA> | **2.3783229** | 3.00E-18 | 150.13146 |
| Seq1 | Nitrososphaeria | Nitrososphaeraceae | <NA> | **7.6266714** | 1.99E-35 | 140.86738 |
| Seq21 | Nitrososphaeria | Nitrososphaeraceae | <NA> | **-1.5967012** | 1.14E-06 | 123.41872 |
| Seq36 | Thermoleophilia | Solirubrobacteraceae | Solirubrobacter | **-4.4758979** | 4.66E-33 | 104.72293 |
| Seq16 | Nitrososphaeria | Nitrososphaeraceae | <NA> | **9.9985217** | 1.55E-23 | 95.99914 |
| Seq24 | <NA> | <NA> | <NA> | **-2.295923** | 9.70E-09 | 93.65713 |
| Seq42 | Alphaproteobacteria | Reyranellaceae | Reyranella | **-2.7515738** | 6.31E-22 | 88.06108 |
| Seq50 | Actinobacteria | Micromonosporaceae | Asanoa | **-0.6003106** | 4.29E-02 | 83.69577 |
| Seq46 | Alphaproteobacteria | Xanthobacteraceae | <NA> | **-2.1148176** | 7.02E-13 | 79.63385 |
| Seq40 | Acidimicrobiia | Ilumatobacteraceae | <NA> | **-2.5855485** | 7.24E-20 | 79.40673 |
| Seq33 | Nitrososphaeria | Nitrososphaeraceae | <NA> | **-7.6638276** | 7.05E-21 | 78.91617 |
| Seq48 | Thermoleophilia | Solirubrobacteraceae | Solirubrobacter | **-1.0144864** | 3.25E-04 | 78.00737 |
| Seq25 | Actinobacteria | Propionibacteriaceae | Microlunatus | **-8.0594616** | 2.26E-18 | 77.47479 |
| Seq81 | Acidimicrobiia | <NA> | <NA> | **-0.8494601** | 4.82E-02 | 74.60126 |
| Seq49 | Gammaproteobacteria | Nitrosomonadaceae | MND1 | **-9.6831806** | 2.15E-20 | 74.20715 |
| Seq53 | Bacteroidia | Chitinophagaceae | <NA> | **9.0250517** | 3.71E-19 | 72.91090 |
| Seq165 | Oxyphotobacteria | <NA> | <NA> | **9.5871242** | 1.07E-11 | 72.14437 |
| Seq119 | Blastocatellia_(Subgroup_4) | Pyrinomonadaceae | RB41 | **6.6721824** | 2.99E-27 | 72.06971 |
| Seq57 | Verrucomicrobiae | Chthoniobacteraceae | Candidatus_Udaeobacter | **-2.5828842** | 4.44E-20 | 71.75281 |
| Seq94 | Subgroup_6 | <NA> | <NA> | **2.0003699** | 8.28E-12 | 67.80215 |
| Seq169 | Nitrososphaeria | Nitrososphaeraceae | <NA> | **1.3963539** | 2.05E-06 | 66.32096 |
| Seq161 | Nitrospira | Nitrospiraceae | Nitrospira japonica | **4.056989** | 3.30E-28 | 65.94252 |
| Seq93 | Gammaproteobacteria | <NA> | <NA> | **-1.5841491** | 1.64E-05 | 65.67189 |
| Seq32 | Nitrososphaeria | Nitrososphaeraceae | Candidatus_Nitrososphaera | **7.6847956** | 1.24E-16 | 62.73745 |
